# The immune landscape of high-grade brain tumor after treatment with immune checkpoint blockade

**DOI:** 10.3389/fimmu.2022.1044544

**Published:** 2022-12-14

**Authors:** Jang Hyun Park, In Kang, Heung Kyu Lee

**Affiliations:** Graduate School of Medical Science and Engineering, Korea Advanced Institute of Science and Technology (KAIST), Daejeon, Republic of Korea

**Keywords:** GBM - Glioblastoma multiforme, PD-1, CD8 T cell, CCL5 - chemokine ligand 5, tumor microenvirenment

## Abstract

Despite the therapeutic success of immune checkpoint blockade (ICB) therapy against multiple tumors, many patients still do not benefit from ICB. In particular, high-grade brain tumors, such as glioblastoma multiforme (GBM), have a very low response rate to ICB, resulting in several failed clinical trials. This low response rate might be caused by a lack of understanding of the unique characteristics of brain immunity. To overcome this knowledge gap, macroscopic studies of brain immunity are needed. We use single cell RNA sequencing to analyze the immune landscape of the tumor microenvironment (TME) under anti-PD-1 antibody treatment in a murine GBM model. We observe that CD8 T cells show a mixed phenotype overall that includes reinvigoration and re-exhaustion states. Furthermore, we find that CCL5 induced by anti-PD-1 treatment might be related to an increase in the number of anti-inflammatory macrophages in the TME. Therefore, we hypothesize that CCL5-mediated recruitment of anti-inflammatory macrophages may be associated with re-exhaustion of CD8 T cells in the TME. We compare our observations in the murine GBM models with publicly available data from human patients with recurrent GBM. Our study provides critical information for the development of novel immunotherapies to overcome the limitations of anti-PD-1 therapy.

## Introduction

Glioblastoma multiforme (GBM) is a devastating high-grade brain tumor that typically results in only 1~2 years of patient survival despite conventional therapy, which includes radiotherapy and chemotherapy (1). Although immune checkpoint blockades (ICBs), such as anti-PD-1 (aPD-1) therapy, have shown efficacy against multiple tumors, their effect on GBM has been disappointing (2), and recent clinical trials of ICBs for GBM have failed (3). Some tumors, including GBM, are classified as “cold tumors” because of their low response rate to ICBs (4). Poor CD8 T-cell infiltration, lack of neo-antigen and neo-antigen spreading, and an immunosuppressive tumor microenvironment (TME) have been proposed as characteristics of cold tumors. Furthermore, immune cell infiltration into the brain parenchyma at the steady state is limited, antigen spreading and drug delivery are inhibited by the blood–brain barrier (BBB), and the dominant proportion of microglia suppresses antitumor immunity (5), making brain tumors especially hard to target with ICB. In addition, a recent study showed that meningeal lymphatics are suppressed in GBM (6). To overcome these hurdles, a comprehensive understanding of the effects of ICB within the brain TME is needed.

Systemic information on the effects of ICB within the brain is lacking. Although most macromolecules are unable to cross the BBB (7), there is evidence that ICB agents can cross the BBB (8), and inflammation induced by antitumor immunity may induce further opening of the BBB (9). Outside the BBB, ICBs have been shown to act on extracranial CD8 T cells to augment their anti-tumor functions and trafficking into the brain (10). ICBs commonly target CD8 T cells, which can directly kill tumor cells. For example, activated CD8 T cells express PD-1, and the resulting PD-1 signaling suppresses their antitumor function; however, when the PD-1 signal is blocked by ICB, the antitumor function of the CD8 T cells is reinvigorated (11). Although not all exhausted T cells respond to ICB, a small fraction of exhausted T cells called precursor exhausted T cells (Tpex) do respond to ICB. Despite that, severe T cell exhaustion may be irreversible (12). In addition, there are reports of PD-1 expression by non-T cells (13–15) and nonspecific activation of macrophages by FcγRs (16), which suggest that ICB may affect multiple cell types and multiple stages of CD8 T cells at various sites, including intracranial and extracranial spaces. Nevertheless, a better understanding of T cell status within the TME of brain tumors is urgently needed to overcome the current limitations of ICB therapies.

To better understand the mechanisms and limitations of ICBs in GBM, bulk analysis of the TME after ICB treatment is indispensable. Therefore, we analyzed the immune landscape of the TME in the presence and absence of aPD-1 treatment using single-cell RNA sequencing (scRNAseq) in a GL261-induced murine GBM model. We observed limited reinvigoration of exhausted CD8 T cells together with increased expression of T-cell exhaustion markers after aPD-1 treatment, which may halt ICB efficacy. Furthermore, we found evidence that CCL5-mediated recruitment of anti-inflammatory macrophages suppressed the reinvigoration of CD8 T cells in the presence of ICB.

## Results

### The immune landscape of anti-PD-1–treated brain tumor tissue

**Figure 1:**
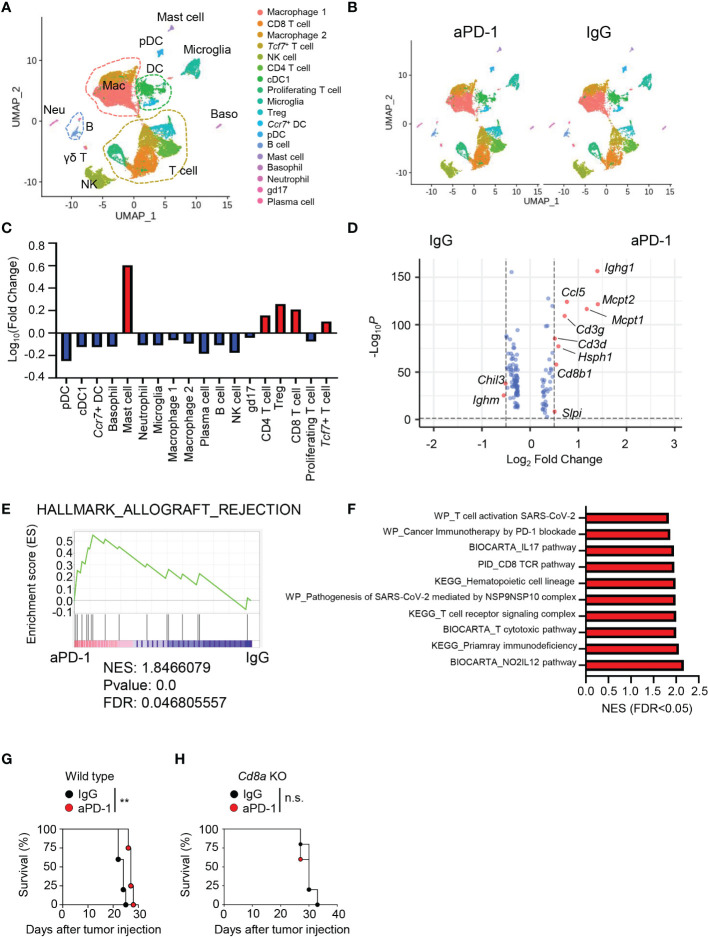
Immune landscape of brain tumor tissue after aPD-1 treatment. **(A–F)** Eight-week-old C57BL/6J mice were intracranially injected with GL261 cells and intraperitoneally injected with aPD-1 at 11, 13, and 15 days post tumor-cell injection. At day 20 after tumor-cell injection, tumor tissues were harvested and analyzed by scRNAseq. **(A)** Data were subjected to UMAP. **(B)** UMAP comparison between aPD-1 and IgG groups. **(C)** Log fold change of clusters after aPD-1 treatment compared with the IgG group was calculated. Red: increased; blue: decreased. **(D)** Volcano plot to visualize DEGs from whole cells of IgG-treated or aPD-1-treated groups. The cutoff fold change was 0.5, and the cutoff *P* value was 0.05. Red dots indicate significantly changed genes. Blue dots indicate non-significant genes. **(E)** DEGs were annotated to HALLMARK_ALLOGRAFT_REJECTION gene sets. **(F)** Gene sets from WikiPathways (WP), BIOCARTA, the Pathway Interaction Database, and KEGG were used to find significantly upregulated pathways. **(G, H)** Wild type (WT) **(G)** or *Cd8a*-knockout **(H)** mice were intracranially injected with GL261 cells. aPD-1 antibody was injected intraperitoneally at 11, 13, and 15 days post tumor-cell injection. Survival was monitored. Survival data were analyzed by log-rank test. *P<0.05, **P<0.01. NES, normalized enrichment score; FDR: false discovery rate. n.s., not significant.

To induce GBM, we injected GL261 cells into C57BL/6J mice. We then treated the mice with three injections of aPD-1 or control immunoglobulin G (IgG). We collected immune cells from the tumor tissues of the mice and performed scRNAseq using the 10X Genomics platform (**Figures S1A, B**). We analyzed a total of 9,037 cells with 18,044 features and 2,000 variable features for the IgG group and 10,895 cells with 18,289 features and 2,000 variable features for the aPD-1 group after filtering. Uniform Manifold Approximation and Projection (UMAP) of the scRNAseq data revealed 18 different clusters of cells (**Figure 1A**), which we identified as the following cell types on the basis of known markers: plasmacytoid dendritic cells (pDCs; *Ccr9, Siglech, Ly6c2*), *Ccr7^+^* DCs (*Ccr7, Flt3, Zbtb46*), basophils (*Gata2, Hdc*), mast cells (*Gata2, Hdc, Mcpt2*), neutrophils (*Ly6g*), macrophage 1 (*Ccr2, Lyz2, Itgam, Apoe*), macrophage 2 (*Ccr2, Lyz2, Itgam*), classical DC 1 (cDC1; *Xcr1, Flt3, Zbtb46, Itgax*), natural killer (NK) cells (*Ncr1*), CD4 T cells (*Cd4, Cd3e*), *Tcf7* ^+^ T cells (*Tcf7, Cd3e*), proliferating T cells (*Mki67, Cd3e*), CD8 T cells (*Cd8a, Cd3e*), regulatory T cells (Tregs; *Foxp3, Cd4, Cd3e*), plasma cells (*Sdc1, Jchain*), B cells (*Ms4a1, Cd19*), microglia (*Siglech, P2ry12, Tmem119, Itgam*), and γδ T (gd17) cells (*Il17a, Trdc*; **Figure S1C**). There were no distinct differences in the UMAP results between the aPD-1 and IgG groups (**Figure 1B** and **Figure S1D**). When we calculated fold changes among the cell types, mast cells had the greatest increase in the aPD-1 group compared with the IgG group, followed by Tregs, CD8 T cells, CD4 T cells, and *Tcf7* ^+^ T cells. On the other hand, pDCs had the greatest reduction in the aPD-1 group, followed by plasma cells, NK cells, and others (**Figure 1C**). Although mast cells showed a dramatic fold change between the groups, their absolute amount was very low in both groups (**Figure S1E**). Therefore, we concluded that T cells were the cells that responded most to aPD-1 therapy, both qualitatively and quantitatively. When we confirmed the scRNAseq results using flow cytometry, we observed that the overall recruitment of immune cells, including CD4 T cells and CD8 T cells, was not significantly affected by aPD-1 (**Figures S1F, G**); however, the number of tumor-specific CD8 T cells was increased by aPD-1 (**Figure S1H**).

To check for qualitative changes induced by aPD-1 treatment, we analyzed differentially expressed genes (DEGs) in all the immune cells. The aPD-1 treatment resulted in more upregulation of genes than downregulation of genes. *Ighm* and *Chil3* were significantly downregulated by aPD-1, whereas *Ighg1, Mcpt1, Mcpt2*, and CD8 T cell–related genes, such as *Cd3g, Cd3d*, and *Cd8b1* were significantly upregulated (**Figure 1D**). Likewise, aPD-1 induced enrichment of allograft rejection–related gene sets, implying enhanced antitumor immunity (**Figure 1E**). In addition, multiple pathways related to T-cell response were upregulated by aPD-1 treatment (**Figure 1F**). Consistent with these findings, aPD-1 treatment extended the survival of GL261-bearing mice (**Figure 1G**); however, the effect was not dramatic, prolonging survival by just a few days. Furthermore, the survival benefit of aPD-1 was abrogated by *Cd8a* deficiency (**Figure 1H**), meaning that the effect of aPD-1 was mediated by CD8 T cells. Taken together, these results indicated that aPD-1 treatment enhanced CD8 T-cell immunity, resulting in a subtle survival benefit.

### CD8 T cells were the immune cells with the most PD-1 expression in the brain TME

**Figure 2:**
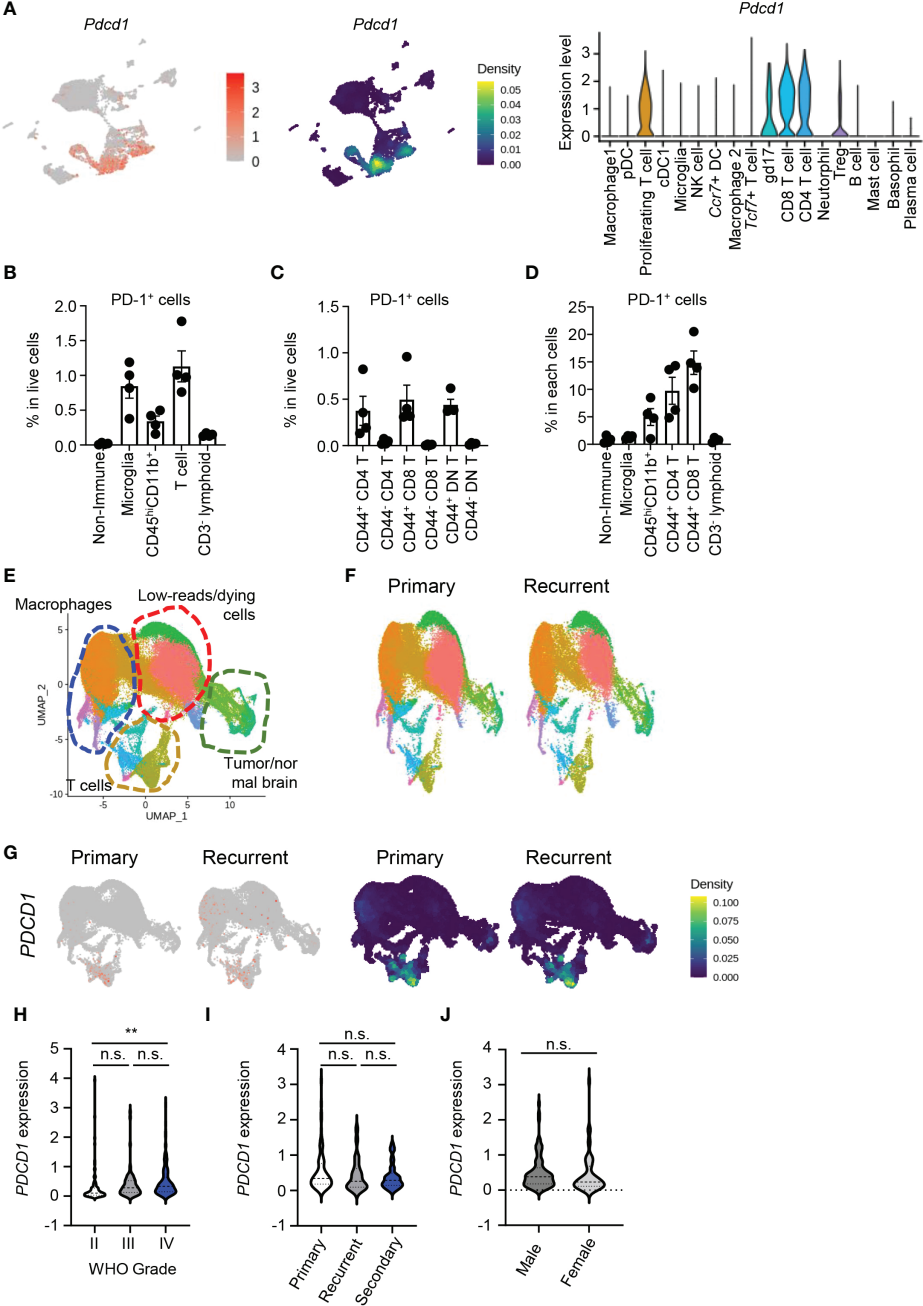
PD-1 is dominantly expressed by CD8 T cells in brain tumor tissues. **(A)** Using scRNAseq data, expression of *Pdcd1* was visualized by FeaturePlot, density plot, and VlnPlot. **(B–D)** Eight-week-old C57BL/6J mice were intracranially injected with GL261 cells, and tumor tissues were analyzed by flow cytometry 10 days later. PD-1^+^ cells among total cells **(B)**, PD-1^+^ T cells among total cells **(C)**, and PD-1^+^ percent from each cell **(D)** were calculated. **(E–G)** Using the GSE154795 dataset, patients with primary (p) and recurrent (r) GBM were analyzed. **(E)** Macrophages, low-reads/dying cells, T cells, and tumor/normal brain cells were identified using markers. **(F)** UMAPs were compared between groups. **(G)** *PDCD1* expression was compared between groups by FeaturePlot and density plot. **(H–J)** Using CGGA data, *PDCD1* expression in patients divided by WHO grade **(H)**, subset of tumor **(I)**, and gender **(J)** were analyzed. Data in **(H–J)** were analyzed by unpaired, two-tailed Student’s *t* test. *P<0.05, **P<0.01. Error bars represent the mean ± standard error of the mean (SEM). n.s., not significant.

Next, we analyzed PD-1–expressing cells to determine direct targets of aPD-1 therapy in the brain TME. When we compared *Pdcd1* expression using the scRNAseq data, *Pdcd1* was dominantly expressed by T cells, including proliferating T cells, gd17 cells, CD8 T cells, CD4 T cells, and Tregs (**Figure 2A**). Among these cell types, CD8 T cells showed the highest expression of *Pdcd1*. Using flow cytometry, we also analyzed the protein levels of PD-1 (**Figure S2A**). Among all cells, T cells had the most PD-1 expression, followed by microglia and CD45^hi^CD11b^+^ cells (**Figure 2B**). Among the T cells, CD8 T cells and CD4 T cells showed similar expression of PD-1. In addition, CD44-expressing effector cells showed dominant expression of PD-1 (**Figure 2C**). The percentage of individual cells expressing PD-1 was highest among the CD8 T cells, followed by the CD4 T cells and the CD45^hi^CD11b^+^ cells (**Figure 2D**).

Next, we evaluated human samples using public data. Using scRNAseq data from patient samples of primary and recurrent GBM (GSE154975) (17), we identified multiple cell types based on markers from the original study: macrophages (*ITGAM*), T cells (*CD3E*), Tregs (*CD4, CD3E, FOXP3*), low-reads/dying cells (*MALAT1*), and tumor/normal brain cells (*GFAP, SOX2*; **Figures S2B** and **Figure 2E**). There were no major differences in the proportions of these cells between primary and recurrent GBM (**Figure 2F**). *PDCD1* expression was dominated by T cells, especially CD8 T cells (**Figure 2G**), and increased as the tumor grade increased (**Figure 2H**). Neither the type of GBM tumor nor patient gender affected *PDCD1* expression (**Figures 2I, J**). Patients with high *PDCD1* expression in their tumors had shorter survival than patients with low *PDCD1* expression in their tumors according to both the Chinese Glioma Genome Atlas (CGGA) (18) and Gene Expression Profiling Interactive Analysis (GEPIA) of The Cancer Genome Atlas (TCGA) (19) (**Figures S2C, D**). These findings indicate that PD-1 is dominantly expressed by CD8 T cells in the brain TME of humans and mice, and PD-1 expression increases with tumor grade and negatively affects patient survival.

### Anti-PD-1 treatment could not stop re-exhaustion of CD8 T cells

**Figure 3:**
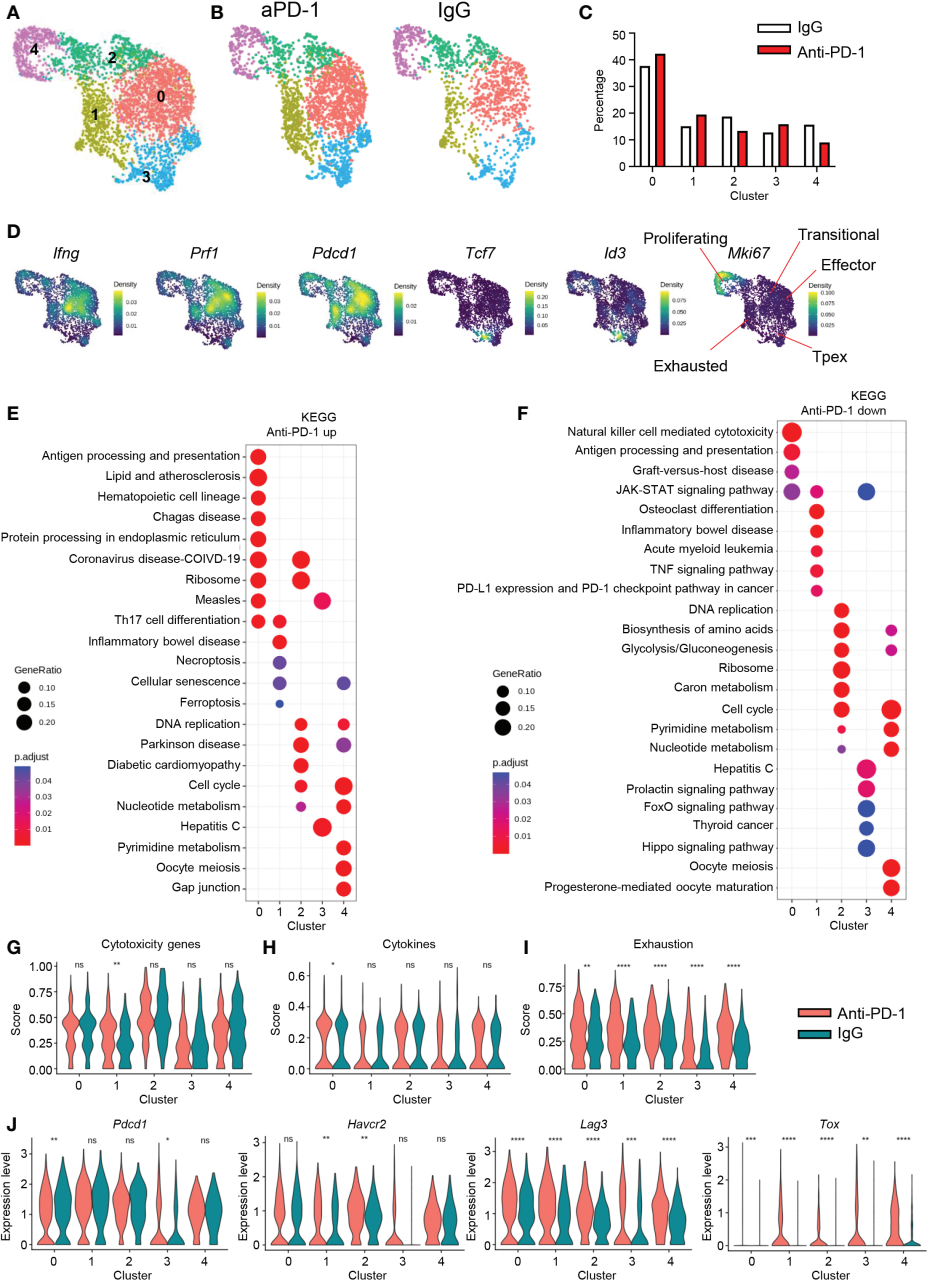
CD8 T-cell signatures after aPD-1 treatment. **(A)** CD8 T cells (*Cd3e^+^Cd8a^+^Cd4^−^* doublet*^−^*) were re-clustered. CD8 T cells are shown by UMAP. **(B, C)** Differences in clusters between IgG and aPD-1 groups are shown by DimPlot **(B)** and bar graph **(C)**. **(D)** Expression densities of *Ifng, Prf1, Pdcd1, Tcf7, Id3*, and *Mki67* are shown with characteristics of clusters. **(E, F)** Pathways that were upregulated **(E)** and downregulated **(F)** by aPD-1 treatment were analyzed using the KEGG database. **(G–I)** Gene scores of clusters were calculated for cytotoxicity genes (*Gzma, Gzmb, Gzmc, Prf1*) **(G)**, cytokine genes (*Ifng, Il2, Tnf*) **(H)**, and exhaustion genes (*Pdcd1, Havcr2, Lag3, Tox, Tigit, Cd160*) **(I)**. **(J)** Expression of *Pdcd1, Havcr2, Lag3*, and *Tox* was analyzed for clusters. Data in **(G–J)** were analyzed by the stat_compare_means function with unpaired t-test. *P<0.05, **P<0.01, ***P<0.001, ****P<0.0001. ns, not significant.

CD8 T cells dominantly expressed PD-1 and were the cell type most affected by aPD-1 treatment. Therefore, we analyzed CD8 T cells from the murine GBM tumors after re-clustering *Cd3e^+^Cd8a^+^Cd4^−^* doublet*^−^* cells. We identified five clusters among the CD8 T cells (**Figure 3A**). After aPD-1 treatment, the overall number of CD8 T cells increased, with no great difference in the composition of each cluster (**Figures 3B, C**); the numbers of cells in clusters 0, 1, and 3 were slightly increased, whereas those in clusters 2 and 4 were reduced. Next, we examined the characteristics of each cluster. Previously, we checked the activation of CD8 T cells using flow cytometry and found that aPD-1 treatment reduced the naïve (CD44*^−^* CD62L^+^) and central memory (CM; CD44^+^CD62L*^−^*) populations, whereas it slightly increased the effector (CD44^+^CD62L*^−^*) population (**Figure S3A**). Multiple genes were differentially expressed among the five CD8 T cell clusters in the murine GBM model (**Figure S3B**). Cluster 0 showed specific expression of heat-shock proteins, *Cd3g*, and *Ccl5*. Cluster 1 showed prominent expression of endosomal sorting complex required for transport (ESCRT)-related genes, such as *Vps37b*, and exhaustion-related *Nr4a* genes. Clusters 2 and 4 were marked by proliferation-related genes, such as *Mki67* and *Stmn1*. Cluster 3 showed expression of naïve-like or precursor-like genes, such as *Tcf7.* We analyzed pathways that were uniquely expressed in the different clusters using the Gene Ontology (GO) (20, 21) and Kyoto Encyclopedia of Genes and Genomes (KEGG) databases (22–24) (**Figures S3C, D**). Cluster 0 showed enrichment of pathways related to protein folding, T-cell receptor (TCR) binding and inflammation. Cluster 1 was characterized by pathways related to transcription and senescence. Cluster 2 was marked by expression of cell cycle-related pathways. Cluster 3 was enriched with viral infection pathways, and cluster 4 was characterized by pathways related to microtubules. In addition, effector molecules such as *Ifng* and *Prf1* were dominantly expressed by cluster 0, whereas cluster 1 showed low expression of *Ifng* and *Prf1* but high expression of *Pdcd1*. On the other hand, cluster 3 showed *Tcf7* and *Id3* expression, and cluster 4 showed *Mki67* expression (**Figure 3D**). Overall, these results revealed multiple clusters of CD8 T cells in the brain TME, with cluster 0 consisting of activated effector cells with endoplasmic reticulum (ER) stress, cluster 1 consisting of exhausted cells, cluster 2 consisting of transitional cells between clusters 4 and 0, cluster 3 displaying properties similar to those of Tpex, and cluster 4 consisting of proliferating cells.

Next, we analyzed the effect of aPD-1 treatment on CD8 T cells by identifying DEGs among the five CD8 T-cell clusters using the KEGG database (**Figures 3E, F**). In cluster 0, aPD-1 treatment resulted in upregulation of genes related to hematopoietic cells, protein process, and ribosomes and downregulation of genes related to NK cell–mediated cytotoxicity. This implies that cluster 0 cells may utilize TCRs rather than NK receptors because of an increase in antigen presentation. Cluster 1 showed increased expression of genes related to cell death and senescence and downregulation of PD-1–PD-L1 interaction, suggesting that cluster 1 is a direct target of aPD-1 and subject to cell death due to robust aPD-1–induced activation. Cluster 2 did not show any distinct change in gene expression after aPD-1 treatment. Cluster 3 showed downregulation of prolactin, FoxO, and the Hippo pathway after aPD-1 treatment, indicating augmented activation (25–27). Cluster 4 showed increased expression of senescence-related genes and decreased expression of metabolic genes after aPD-1 treatment. To assess functional changes in the CD8 T cells after aPD-1 treatment, we analyzed cytotoxicity-related genes. Although we observed trends of *Gzma, Gzmb*, and *Gzmc* downregulation after aPD-1 treatment, *Prf1* was strongly upregulated, resulting in significant enhancement of cytotoxicity feature for the cluster 1 cells (**Figure S3E** and **Figure 3G**). Furthermore, there was a large increase of *Ifng* expression in all the clusters (**Figure S3F**), and cytokine genes were highly upregulated in cluster 0 after aPD-1 treatment (**Figure 3H**). Therefore, we concluded that aPD-1 treatment directly reinvigorated cluster 1 cells and increased their cytotoxicity. In addition, aPD-1 may enhance TCR ligation and cytokine secretion from cluster 0 cells.

Because the purpose of aPD-1 therapy is reinvigoration of immune cells through suppression of immune cell exhaustion, we tested the effects of aPD-1 on exhaustion markers (*Pdcd1, Havcr2, Lag3, Tox, Tigit*, and *Cd160*). Unexpectedly, the exhaustion score for every CD8 T-cell cluster was increased by aPD-1 therapy (**Figure 3I**). Although *Pdcd1* expression was downregulated in cluster 0, it was increased in cluster 3. In addition, the expression of other exhaustion markers, especially *Lag3* and *Tox*, was upregulated by aPD-1 treatment (**Figure 3J**). The expression of *Tigit* and *Cd160* was very low compared with that of other exhaustion markers (**Figure S3G**). These results imply that augmentation of CD8 T cells by aPD-1 induces expression of exhaustion markers other than PD-1, which may be a direct result of aPD-1 exposure or a compensatory response to the suppression of PD-1 signaling.

Because cluster 1 seemed to directly respond to aPD-1, we also identified DEGs between cluster 1 and the other clusters. In cluster 1, DEGs such as *Nr4a3*, *Vps37b*, *Crem*, and others might be markers of aPD-1-responding CD8 T cells (**Figure S3H**). In addition, we identified gene sets that were most affected by aPD-1 in cluster 1 (**Figure S3I**). Overall, the transcriptional changes among the five CD8 T-cell clusters after aPD-1 treatment suggest that aPD-1 therapy affects a small fraction of CD8 T cells by enhancing their cytotoxicity and cytokine production, although re-exhaustion features were confounded.

### CD4 T-cell signatures after aPD-1 treatment

**Figure 4:**
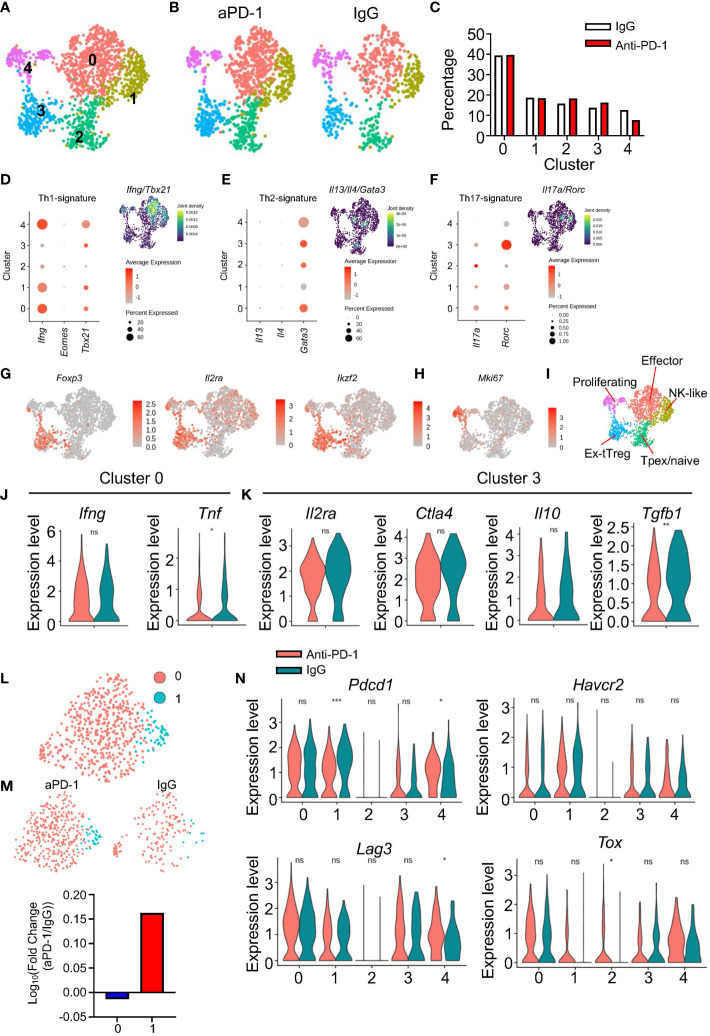
Immunological features of CD4 T cells after aPD-1 treatment. **(A)** CD4 T cells (*Cd3e^+^Cd4^+^Cd8a^−^* doublet*^−^*) were re-clustered. CD4 T-cell clusters are shown by UMAP. **(B, C)** Changes in clusters of CD4 T cells between IgG and aPD-1 groups are shown by DimPlot **(B)** and bar graph **(C)**. **(D–F)** Th1 signature genes (*Ifng, Eomes, Tbx21*) **(D)**, Th2 signature genes (*Il13, Il4, Gata3*) **(E)**, and Th17 signature genes (*Il17a, Rorc*) **(F)** were analyzed. **(G, H)** Expression of *Foxp3, Il2ra, Ikzf2* **(G)**, and *Mki67* **(H)** from each cluster is shown by FeaturePlot. **(I)** Characteristics of clusters of CD4 T cells were designated. **(J)** Gene expression of *Ifng* and *Tnf* in cluster 0 is shown. **(K)** Gene expression of *Il2ra*, *Ctla4*, *Il10*, and *Tgfb1* in cluster 3 was analyzed. **(L, M)** Tregs were sub-clustered. **(L)** Two clusters were identified. **(M)** Differences of clusters in Tregs between IgG and aPD-1 groups are shown. **(N)** Expression of *Pdcd1, Havcr2, Lag3*, and *Tox* in each cluster was analyzed. Data in **(J**, **K, N)** were analyzed by the stat_compare_means function with unpaired t-test *P<0.05, **P<0.01, ***P<0.001. ns, not significant.

Next, we checked how CD4 T cells responded to aPD-1 treatment. Prior to scRNAseq analysis, we checked the activation status of CD4 T cells based on expression of CD44 and CD62L. Unlike the CD8 T cells, the overall activation status of CD4 T cells was not changed by aPD-1 treatment (**Figure S4A**). When we re-clustered *Cd3e^+^Cd4^+^CD8a^−^* doublet*^−^* cells, we identified five different clusters (**Figure 4A**). The proportions of CD4 T cells in each cluster were not affected by aPD-1 treatment (**Figures 4B, C**). Using DEG analysis, we identified marker genes for each CD4 T-cell cluster (**Figure S4B**). Cluster 0 showed expression of activation markers, such as *Nrp1* and *Tnfsf8.* Cluster 1 showed *Ifitm1*, *Cxcr6*, and *Klrd1* expression, resembling cytotoxic cells. Cluster 2 showed *S1pr1, Tcf7, Klf2* expression, implying that this cluster is composed of naïve cells or Tpex. Cluster 3 was marked by *Foxp3* expression, which identified those cells as Tregs. Cluster 3 cells expressed *Ikfzf2*, implying that they were thymic Tregs (28). Cluster 4 was characterized by the expression of cell cycle–related genes. When we analyzed expression of T helper 1 (Th1) signature genes in the CD4 T-cell clusters, cluster 0 showed the highest expression of *Ifng* and *Tbx21*; however, *Eomes* expression was rarely detected (**Figure 4D**). We also evaluated Th2 genes in the CD4 T cells and found that although *Gata3* was expressed ubiquitously, *Il13* and *Il4* expression was very low (**Figure 4E**). Next, we checked the expression of Th17 genes in the CD4 T-cell clusters and found that *Il17a* was expressed in cluster 2 and *Rorc* expression was highly enriched in cluster 3; however, the Th17 gene set density was most enriched in cluster 0 (**Figure 4F**). These data suggest that cluster 0 is composed of activated CD4 T cells, including various subsets of Th cells with a dominant proportion of Th1 cells. Interestingly, cluster 3, which was identified as thymic Tregs, showed *Rorc* expression.

When we plotted Treg markers using the FeaturePlot function, we observed that *Foxp3* expression was aggregated in cluster 3 of the CD4 T cells, whereas *Il2ra* and *Ikzf2* expression was relatively scattered among the clusters (**Figure 4G**). Cluster 1 showed expression of NK receptors, such as *Klrk1* and *Klrd1*; however, the cluster 1 cells did not express *Ncr1* (**Figure S4C**) or *Prf1* (**Figure S4D**). These data indicate that cluster 1 of the CD4 T cells is composed of NK-like cells, but not NK T cells or cytotoxic CD4 T cells. We used the GO and KEGG databases to further identify the other clusters (**Figures S4E, F**). Cluster 0 showed expression of multiple gene sets involved in interactions with other cells and Th differentiation. In addition, cluster 0 showed enrichment of PD-1–PD-L1 interaction genes, implying that cluster 0 cells are possible responders to aPD-1. Cluster 1 was enriched with inhibitor-activity genes and NK-mediated cytotoxicity genes. Cluster 2 was marked by expression of cell adhesion genes. Cluster 3 was characterized by expression of GTP-related genes and IgA-related genes. Cluster 4 showed enrichment of cell cycle–related genes. Furthermore, cluster 4 consistently showed high expression of *Mki67* (**Figure 4H**). Thus, we classified the five CD4 T-cell clusters as follows: cluster 0, effectors; cluster 1, NK-like cells; cluster 2, Tpex/naïve cells; cluster 3, thymic Tregs; cluster 4, proliferating cells (**Figure 4I**).

Because we could not detect any meaningful differential regulation of pathways among the CD4 T-cell clusters in response to aPD-1, we analyzed individual genes. After aPD-1 treatment, cluster 0 showed decreased *Tnf* expression with no change in *Ifng* expression (**Figure 4J**). This suggests that aPD-1 does not strongly affect effector CD4 T cells. Next, we evaluated Treg functions. There were no significant changes after aPD-1 treatment except for a decrease of *Tgfb1* expression (**Figure 4K**). When we further analyzed Tregs by sub-clustering, two different clusters were identified (**Figure 4L**). The aPD-1 treatment increased the proportion of sub-cluster 1 (**Figure 4M**), which was characterized by *Mki67* expression (**Figure S4G**) and expression of cell cycle–related gene sets (**Figure S4H**). This suggests that although aPD-1 did not affect the suppressive function of Tregs, it enhanced Treg proliferation and maintenance. On the other hand, *Mki67* expression in cluster 4 was not affected by aPD-1 (**Figure S4I**).

Clusters 0, 1, 3, and 4 of the CD4 T cells showed robust expression of exhaustion markers. The aPD-1 treatment reduced *Pdcd1* expression in clusters 1 and 4; however, *Havcr2* expression was not affected, and *Lag3* expression was increased only in cluster 4 (**Figure 4N**). Likewise, *Tox* expression was not significantly changed by aPD-1 treatment, except in cluster 2. These data suggest that aPD-1 did not affect the exhaustion of CD4 T cells as much as it affected the exhaustion of CD8 T cells. Furthermore, although cluster 0 showed the possibility to be targeted by aPD-1 antibody, treatment with the antibody did not result in any functional changes; however, the number of proliferative Tregs was increased by aPD-1 therapy.

### Macrophages were preferentially affected by aPD-1 treatment compared with microglia

**Figure 5:**
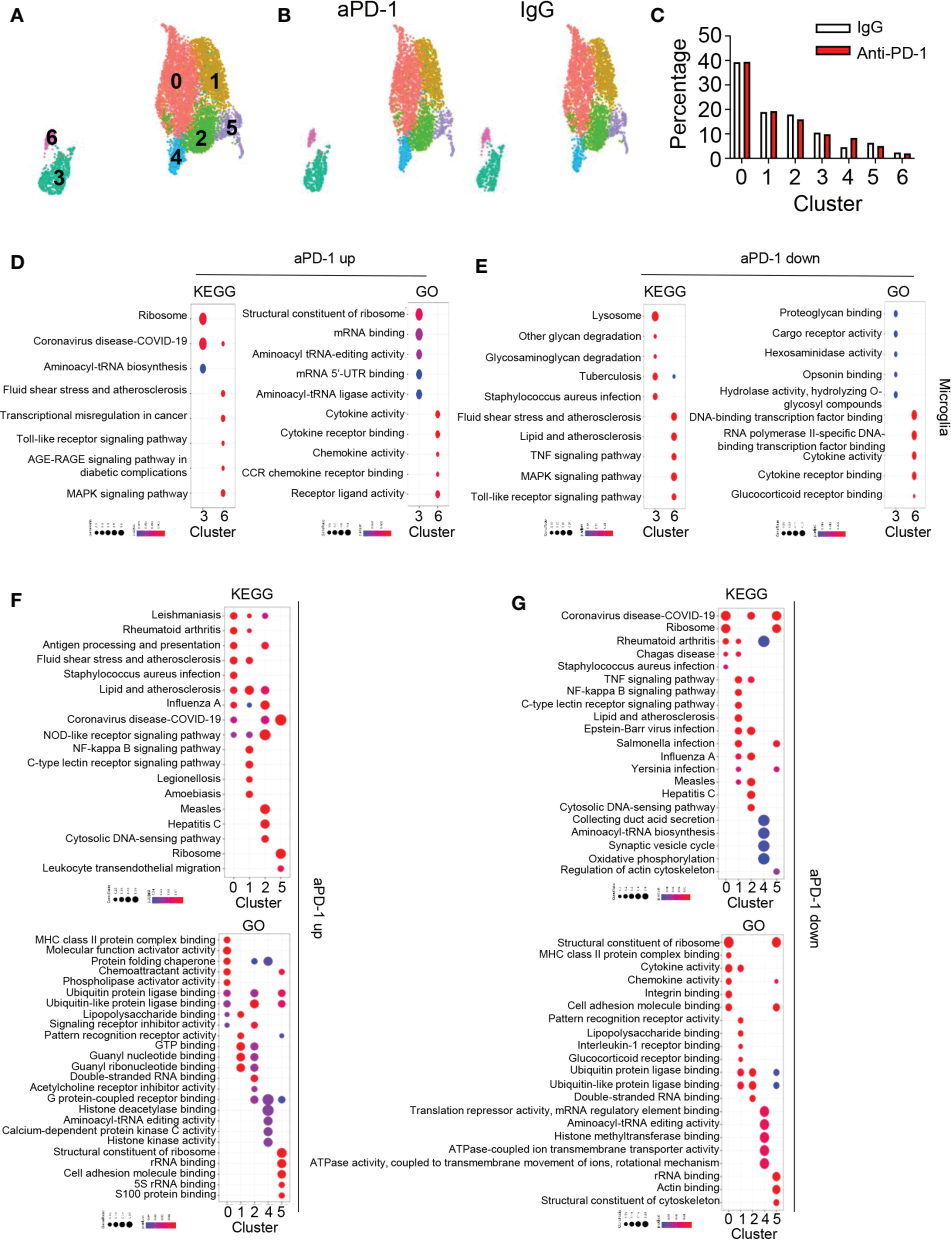
aPD-1–mediated alteration of microglia and macrophage transcriptomes. **(A)** Microglia, macrophage 1, and macrophage 2 clusters from **Figure 1** were isolated and re-clustered. Seven different clusters were identified. **(B, C)** Changes in clusters of microglia and macrophages are shown by DimPlot **(B)** and bar graph **(C)**. **(D, E)** Dot plots for KEGG and GO pathways that were upregulated **(D)** or downregulated **(E)** by aPD-1 treatment in microglia (clusters 3 and 6). **(F, G)** Dot plots for KEGG and GO pathways that were upregulated **(F)** or downregulated **(G)** by aPD-1 treatment in macrophage clusters (clusters 0, 1, 2, 4, and 5). ns, not significant.

Next, we analyzed macrophages and microglia because they are the predominant cell types in the healthy brain and brain tumor tissues. We observed seven different clusters among these cells (**Figure 5A**). There were no great changes in the composition of the clusters after aPD-1 treatment (**Figures 5B, C**). To characterize the clusters, we analyzed DEGs among them (**Figure S5A**). Clusters 3 and 6 were composed of microglia expressing *Tmem119*, *P2ry12*, and *Cx3cr1*. Cluster 3 showed higher expression of microglia signature genes than cluster 6, which means that cluster 6 is composed of inflammatory microglia and cluster 3 is composed of homeostatic microglia. Among the macrophage clusters, cluster 0 showed expression of MHC-related genes, implying that it is composed of antigen-presenting cells. Cluster 1 expressed *Clec4e* and *Nlrp3*, indicating that the cells in cluster 1 can sense pathogen-associated molecular patterns (PAMPs). Clusters 2 and 5 expressed *Ly6c*. Cluster 4 seemed to be composed of low-reads and dying cells because the cells in that cluster expressed common genes at lower levels than the cells in the other clusters. Thus, clusters 2 and 5 seem to contain monocytes. In contrast to cluster 2, cluster 5 showed expression of neutrophil-associated genes such as *Cd177* and *Ace*. To further understand the different roles of the seven clusters, we analyzed pathways using the GO and KEGG databases (**Figures S5B, C**). Cluster 0 showed enrichment of pathways related to immune cell interactions (e.g., cytokine activity) and antigen presentation. Cluster 1 was enriched with pathways involved in PAMP sensing and C-type lectin receptors. Cluster 2 expressed pathways related to RNA sensing and NOD-like receptor sensing. Cluster 3 showed expression of immune reaction–related pathways, such as lysosome, complement, and coagulation cascades. Clusters 4 and 5 did not show distinct features. Cluster 6 was enriched with expression of genes related to the AGE-RAGE signaling pathway. In addition, cluster 0 contained antigen-presenting macrophages, cluster 1 contained PAMP-sensing macrophages, and clusters 2 and 5 contain monocytes.

Cluster 6 showed expression of multiple genes associated with other cells. For example, *Nav3* is commonly expressed by neurons and oligodendrocytes, and *Pmp22* is usually expressed by Schwann cells (29, 30). Therefore, we assumed that the cells in cluster 6 participate in synapse pruning or phagocytosis of other cells. Microglia dominantly express *Mertk*, which is related to synapse engulfment (31). On the other hand, expression of *Axl* was low in cluster 6 (**Figure S5D**). The microglia and the cluster 0 macrophages expressed *Trem2*, which is a marker for disease-associated microglia (**Figure S5E**) (32); however, representative markers for other cell types (e.g., *Rbfox3* for neurons, *Gfap* for astrocytes, and *Olig1* for oligodendrocytes) were not detected (**Figure S5F**). Therefore, we concluded that microglia may not phagocytose other central nervous system–associated cells.

Next, we analyzed pathways affected by aPD-1 therapy. First, we evaluated pathways of microglia using the KEGG and GO databases (**Figure 5D**). Homeostatic microglia (cluster 3) showed a greater increase in expression of translation-related pathways than reactive microglia (cluster 6). On the other hand, reactive microglia showed greater enhancement of immune activation-related pathways. The aPD-1 treatment downregulated glycan-related pathways and opsonin-related pathways in homeostatic microglia, but it downregulated glucocorticoid receptor signaling and transcriptional regulation in reactive microglia (**Figure 5E**). In macrophages, aPD-1 treatment upregulated antigen presentation–related pathways in cluster 0, GTP-related pathways and NOD-like receptor signaling pathways in cluster 1, cytosolic DNA–sensing pathways in cluster 2, and migration and adhesion pathways in cluster 5 (**Figure 5F**). By contrast, aPD-1 downregulated ribosome-related pathways and adhesion in cluster 0 and lipopolysaccharide binding and multiple infection-associated genes in cluster 1. These data suggest that PAMP sensing by the cluster 1 cells is downregulated by aPD-1 except for sensing by NOD-like receptors. Furthermore, cluster 2 showed a decrease of double-stranded RNA binding, and cluster 5 showed a decrease in cytoskeleton functions (**Figure 5G**). These results indicate that aPD-1 regulates multiple aspects of microglia and macrophages; however, it preferentially induces activation of macrophages rather than activation of microglia.

We also analyzed expression of individual antitumor immunity–associated genes in the clusters. Cytokines such as *Il6*, *Il12a*, and *Il12b* were not actively detected (**Figure S5G**). The T-cell chemoattractant *Cxcl9* was upregulated by aPD-1 in clusters 0, 2, and 4, and *Cxcl10* expression was reduced in cluster 2 (**Figure S5H**). Furthermore, *Cd80* was upregulated by aPD-1 in cluster 1, and *Cd86* expression was reduced in cluster 3 (**Figure S5I**). Although *Il10* was detected only at low levels, robust *Tgfb1* expression was observed in multiple clusters. The aPD-1 treatment significantly reduced *Tgfb1* expression; however, it upregulated *Cd274* expression in clusters 0 and 1 (**Figure S5J**). Thus, aPD-1 induced an overall mixed phenotype with expression of both anti- and pro-tumoral genes. These data may be consistent with weak activation accompanying re-exhaustion of CD8 T cells.

### *Ccl5* recruits anti-inflammatory macrophages

**Figure 6:**
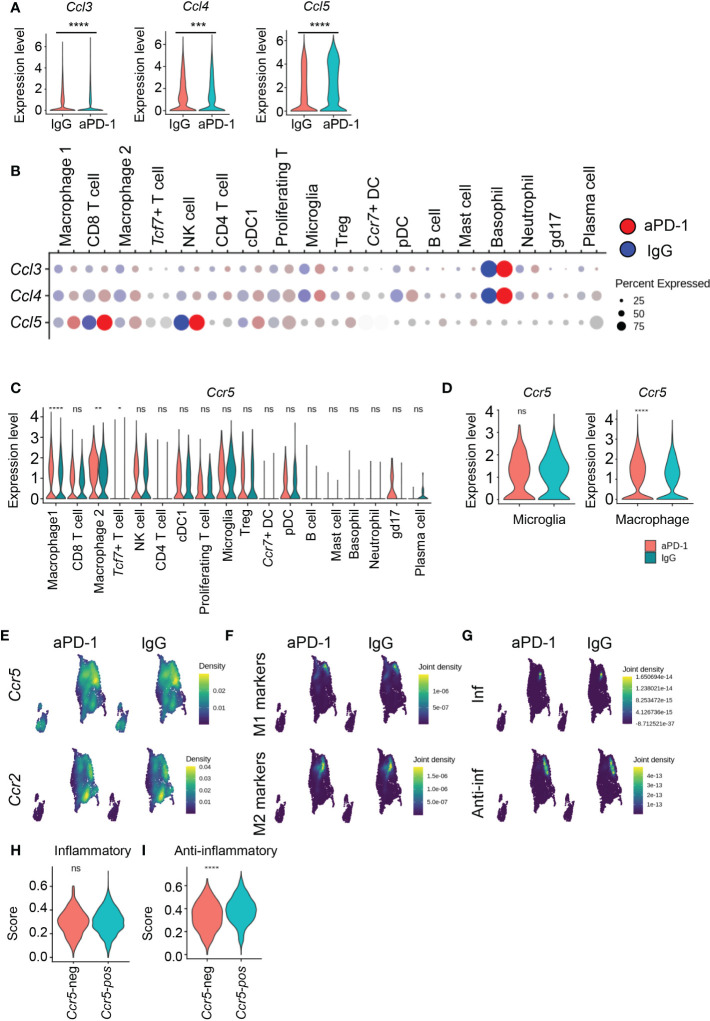
aPD-1-induced *Ccl5* recruits *Ccr5^+^* anti-inflammatory macrophages. **(A)** The expression of *Ccl3*, *Ccl4*, and *Ccl5* from whole immune cells from IgG-treated and aPD-1-treated groups. **(B)** Expression of *Ccl3*, *Ccl4*, and *Ccl5* in different immune cells of IgG-treated or aPD-1-treated groups. **(C, D)** Expression of *Ccr5* in different immune cells **(C)** and microglia and macrophages **(D)**. **(E)** The expression density of *Ccr5* and *Ccr2* from IgG-treated or aPD-1-treated macrophages/microglia. **(F)** The expression density of M1 markers (*Nos2*, *Itgax*, *Cd80*, *Cd86*) and M2 markers (*Tgfb1*, *Il10*, *Arg1*, *Mrc1*) from IgG-treated or aPD-1-treated macrophages/microglia. **(G)** The expression density of inflammatory (*Cd86*, *Cd80*, *Cxcl10*, *H2-Ab1*, *Il1b*, *Tlr2*, *Tnf*, *Il6*, *Il12a*, *Nos2*, *H2-DMb2*) and anti-inflammatory (*Tnfbi*, *Il1rn*, *Il10rb*, *Cd274*, *Il4ra*, *Msr1*, *Tgfb1*, *Il6st*) genes from IgG-treated or aPD-1-treated macrophages/microglia. **(H, I)** The expression of inflammatory **(H)** and anti-inflammatory **(I)** genes in *Ccr5-*negative or *Ccr5-*positive macrophages. Data were analyzed by the stat_compare_means function with unpaired t-test. *P<0.05, **P<0.01, ***P<0.001, ****P<0.0001. ns, not significant.

We next asked why CD8 T cells were re-exhausted even after aPD-1 treatment (**Figure 3I**). Among the DEGs that were upregulated in immune cells by aPD-1, we detected *Ccl5* among the genes known to be associated with CD8 T cells and mast cells (**Figure 1D**). We evaluated expression of CCR5 ligands and found that although *Ccl3* and *Ccl4* were downregulated by aPD-1 treatment, *Ccl5* expression was strongly augmented by aPD-1 (**Figure 6A**). *Ccl3* and *Ccl4* were dominantly expressed by basophils, whereas *Ccl5* was preferentially expressed by CD8 T cells and NK cells (**Figure 6B**). Because the overall *Ccl5* expression level was higher than the overall *Ccl3* and *Ccl4* expression levels and the number of CD8 T cells was greater than the number of basophils, it is possible that the increase in *Ccl5* expression due to aPD-1 treatment may have overcome the reduction of *Ccl3* and *Ccl4* expression due to the same treatment. The total amount of *Ccr5* expression was not increased by aPD-1 (**Figure S6A**); however, *Ccr5* expression by macrophages was increased by aPD-1 (**Figure 6C**). Interestingly, *Ccr5* expression in microglia was not affected by aPD-1 (**Figure 6D**). These data suggest selective recruitment of *Ccr5*-expressing cells in response to aPD-1 treatment.

Among the macrophage/microglia clusters, *Ccr5* expression was enriched in cluster 1, but *Ccr2* was mostly concentrated in cluster 2 (**Figure 6E**), suggesting differences in chemotaxis among monocyte populations. Next, we evaluated the expression of M1 and M2 markers (**Figure S6B**). There was no difference in expression of M1 markers among clusters after aPD-1 treatment, whereas expression of M2 markers was increased by aPD-1 in clusters 0 and 5 and reduced in cluster 2 (**Figure S6C**). These data suggest that M2 markers were preferentially affected by aPD-1. In addition, M2 markers were enriched in *Ccr5*-expressing cells, although M1 markers were also detected in *Ccr5*-expressing cells (**Figure 6F**). These results may be a reflection of the heterogeneity of macrophages in the TME, which makes it difficult to divide the macrophages into M1 and M2 subsets. Therefore, we evaluated inflammatory and anti-inflammatory genes that were previously shown to be related to the response rate to aPD-1 (33). Inflammatory genes were downregulated by aPD-1 in cluster 0 (**Figures S6D, E**). The expression of anti-inflammatory genes was not affected by aPD-1 in clusters 0 and 1; however, it was reduced in cluster 5 (**Figures S6F, G**). Expression of inflammatory genes was also detected in cluster 1, although broad expression of anti-inflammatory genes was also detected in cluster 1, resulting in overlap with *Ccr5* expression (**Figure 6G**). Cluster 0 showed expression of genes related to antigen presentation (**Figure S5A**) but did not show any expression of M1 markers or inflammatory genes. Rather, cluster 1 showed mixed phenotypes. These data imply that cluster 1 contains tumor-associated macrophages that participate in immune reactions, especially as expression of M2 markers and anti-inflammatory genes overlapped with *Ccr5* expression. When we divided the macrophages on the basis of *Ccr5* expression, *Ccr5* ^+^ macrophages showed an increase in anti-inflammatory gene score without a change in inflammatory gene score (**Figures 6H, I**). Thus, the *Ccl5*–*Ccr5* interaction of macrophages may induce preferential accumulation of anti-inflammatory and M2-like macrophages in the brain TME. On the other hand, proliferating Tregs did not express *Ccr5*, and the *Ccr5* expression level was not affected by aPD-1 (**Figure S6H**).

Despite the enhancement of anti-inflammatory features, we did not observe an increase in expression of inhibitory cytokines, such as *Il10* and *Tgfb1* (**Figure S5J**). Although an increase of *Cd274* expression may be critical for re-exhaustion of CD8 T cells, we tried to find additional candidate genes. Interestingly, in clusters 0 and 1, aPD-1 treatment increased *Arg1* expression (**Figure S6I**). Arg1 is an M2 marker known to suppress neighboring immune cells *via* amino acid deprivation (34, 35). The representative pathway induced by L-Arg starvation in CD8 T cells is cell-cycle arrest due to phospho-GCN2-mediated downregulation of D-type cyclins and *Cdk4* (36). When we analyzed expression of *Ccnd3* and *Cdk4* in CD8 T cells, we found that it was globally downregulated by aPD-1 (**Figure S6J**). *Mki67* expression in the proliferating cells of cluster 4 was also downregulated by aPD-1 (**Figure S6K**). GO analysis revealed downregulation of multiple pathways related to translation and transcription after aPD-1 treatment in CD8 T cells (**Figure S6L**). Therefore, we propose that accumulation of anti-inflammatory macrophages suppresses CD8 T cells *via* amino acid deprivation.

### *CCL5* mediated re-exhaustion of CD8 T cells in human patients with recurrent GBM

**Figure 7:**
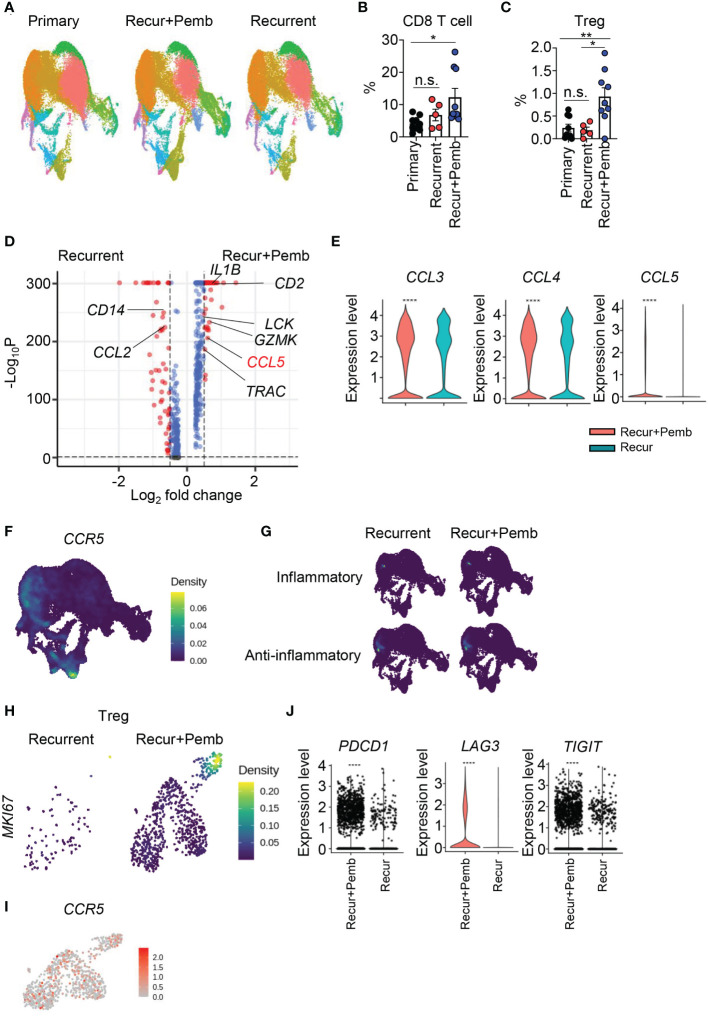
Neoadjuvant pembrolizumab treatment in patients with recurrent GBM induces the *CCL5*–*CCR5* axis. **(A–I)** scRNAseq data from the GSE154795 dataset of tumor tissues from patients with primary GBM, recurrent GBM, and recurrent GBM+pembrolizumab were re-analyzed. **(A)** Cells were subjected to UMAP, and differences in clusters are displayed. Frequencies of CD8 T cells **(B)** and Tregs **(C)** among total cells were calculated. DEGs in total cells between patients with recurrent GBM and patients with recurrent GBM+pembrolizumab were analyzed. **(E)** The expression of *CCL3*, *CCL4*, and *CCL5* in total cells was compared between patients with recurrent GBM and patients with recurrent GBM+pembrolizumab. Expression densities of *CCR5* **(F)** and inflammatory genes (*CD86*, *CD80*, *CXCL10*, *IL1B*, *TLR2*, *TNF*, *IL6*, *IL12A*, *NOS2*) and anti-inflammatory genes (*TGFBI*, *IL1RN*, *IL10RB*, *CD274*, *IL4R*, *MSR1*, *TGFB1*, *IL6ST*) **(G)** are shown. **(H)** The expression density of *MKI67* in Tregs. **(I)** Feature plot for *CCR5* expression of Tregs. **(J)** The expression of *PDCD1, LAG3*, and *TIGIT* in CD8 T cells was analyzed. Data in **(B, C)** were analyzed by unpaired, two-tailed Student’s *t* test. Data in **(E**, **J)** were analyzed by the stat_compare_means function with unpaired t-test. *P<0.05, **P<0.01, ***P<0.001, ****P<0.0001. Error bars represent the mean ± standard error of the mean (SEM). ns, not significant.

To evaluate the clinical significance of our data, we compared our data to public scRNAseq data from patients with primary (p) or recurrent (r) GBM (GSE154795) (17). Some patients with rGBM had been treated with neoadjuvant pembrolizumab (rGMB+pemb). We subjected the human samples to UMAP and identified cell clusters (**Figure 7A**) that were subsequently characterized on the basis of multiple markers (**Figure S2B**). The frequency of CD8 T cells was increased by pembrolizumab (pemb) treatment, as expected (**Figure 7B**); however, the number of Tregs was also increased (**Figure 7C**). We analyzed DEGs between the patients with rGBM and the patients with rGBM+pemb. Consistent with our previous data (**Figure 6**), the patients with rGBM+pemb had reduced *CCL2* expression and increased expression of *CCL5* and other T cell–related genes (e.g., *TRAC* and *LCK*) compared with the patients with rGBM (**Figure 7D**). In addition, expression of *CCL3*, *CCL4*, and *CCL5* was increased by pemb (**Figure 7E**). Although CD8 T cells showed the highest expression of *CCR5*, Tregs and macrophage/microglia also showed modest expression of *CCR5* (**Figure 7F**). We also evaluated inflammatory and anti-inflammatory genes. Like the murine data, expression of the anti-inflammatory gene set overlapped with *CCR5* expression in macrophages/microglia (**Figure 7G**). In addition, the numbers of *Mki67* ^hi^ proliferating Tregs were increased by pemb treatment (**Figure 7H**). However, in contrast to the murine data, proliferative Tregs in the human samples robustly expressed *CCR5* (**Figure 7I**). As a result, multiple exhaustion markers, including *PDCD1*, *LAG3*, and *TIGIT*, were upregulated by pemb in the human patients (**Figure 7J**), though increases of *TOX* and *HAVCR2* expression were not significant (**Figure S7A**). Also, in accordance with the murine data, pemb treatment in humans reduced expression of *CDK4* in CD8 T cells (**Figure S7B**).

Analysis of CGGA data revealed that *CCL5* expression was positively correlated with *PDCD1*, *HAVCR2*, and *LAG3* expression (**Figure S7C**). Thus, *CCL5*-mediated re-exhaustion may be conserved in both humans and mice. In addition, *CCL5*, *HAVCR2*, and *LAG3* expression was not different between patients who received neoadjuvant pemb treatment and those who received adjuvant pemb treatment (GSE121810) (37); however, *PDCD1* expression was slightly higher in the patients who received neoadjuvant pemb (**Figure S7D**). Taken together, our results suggest that *CCL5* may be related to re-exhaustion of CD8 T cells in human patients (**Figure S8**), and this axis does not differ between neoadjuvant and adjuvant pemb treatment.

## Discussion

Although a better understanding of how aPD-1 works in the brain TME at a macroscopic level is urgently needed, relevant data are lacking. We analyzed scRNAseq data from untreated and aPD-1-treated GL261-bearing mice and found that aPD-1 facilitated tumor infiltration of CD8 T cells, which could be divided into five different subsets. Among them, one subset displayed an enhanced cytotoxicity gene score in response to aPD-1 treatment, indicating that those cells may be a direct target of aPD-1. Furthermore, we identified aPD-1-responding gene sets in CD8 T cells and their transcriptomic changes in response to aPD-1. We also found that expression of exhaustion markers rebounded after aPD-1 treatment, which may be a reason for the limited efficacy of aPD-1 and clinical trial failure. Proliferative Tregs and anti-inflammatory macrophages accumulated in response to aPD-1 therapy *via* the *Ccl5*–*Ccr5* interaction. Furthermore, *Cd274* and *Arg1* were upregulated after aPD-1 treatment in macrophages. The increase of *Arg1* expression was associated with reduced transcription and translation activity in CD8 T cells. We confirmed these results from the murine GBM model using scRNAseq data from patients with rGBM. Our data provide critical information for the development of novel therapeutic targets and strategies to overcome the limitations of anti-PD-1 therapy in patients with GBM.

We showed that aPD-1 therapy had a subtle survival benefit in GL261-beraing mice that depended on CD8 T cells. However, other studies have shown that aPD-1 may directly affect other immune cells, such as macrophages, *via* Fc receptors (FcRs) (16). Heimberger and colleagues previously showed that aPD-1 was effective in *Cd8a*-deficient mice, providing a survival benefit mediated by macrophages. The different mechanisms of aPD-1 benefits may be related to the different experimental systems used, as different tumor models can have different antitumor immune responses (38). In addition, Heimberger and colleagues injected mice with aPD-1 three times weekly for up to 5 weeks, whereas we only injected mice with aPD-1 three times in total. We suspect that repeated injection may increase the chance for aPD-1 to be recognized by FcRs, and this possibility should be tested in the future. Furthermore, PD-1 expression in macrophages has been reported (13). Our data show PD-1 expression in microglia and macrophages; however, the expression of PD-1 in macrophages was lower than that in CD8 T cells. Moreover, there was no detectable *Pdcd1* expression in macrophages and microglia in the mouse and human scRNAseq data. It is possible that macrophage and microglia PD-1 expression in brain tumors is overstated because of the large overall numbers of those cells in the brain TME. Because we clearly detected a CD8 T cell cluster in which PD-1 signaling was downregulated after aPD-1 treatment, we hypothesize that CD8 T cells may be the target of aPD-1 in the brain TME in our system.

Cluster 1 of CD8 T cells were responders to aPD-1. These cells expressed *Nr4a3* and *Nr4a2*, which are known to establish a positive feedback loop with TOX (39). Cluster 1 also showed high expression of *Pdcd1* without corresponding expression of functional genes, such as *Ifng*. Therefore, we hypothesized that cluster 1 is composed of exhausted CD8 T cells. A recent study showed that Tpex are genuine responders to aPD-1 (12). Our cluster 3 of CD8 T cells expressed the Tpex markers *Tcf7* and *Id3* (40); however, we did not observe any significant change in cluster 3 after aPD-1 treatment. Thus, our data suggest that exhausted CD8 T cells, rather than Tpex, may be the most likely target of aPD-1 in the brain TME. On the other hand, aPD-1 treatment may affect the progression of T-cell exhaustion, so it is possible that we misidentified cluster 1 as responder cells because of distortion of the exhaustion process. Therefore, although our data provide information implicating a possible population of aPD-1–responding cells, these results should be further qualified. In any event, our DEG data comparing cluster 1 in untreated and aPD-1-treated mice can be used to identify markers to predict responders and nonresponders to ICB therapy.

Tregs were not substantially affected by aPD-1 treatment, although aPD-1 did increase the size of the proliferative subset of Tregs. Therefore, although the expression of immune-suppressive genes was not affected by aPD-1, the enhanced proliferative capacity of Tregs could provide sustained immunosuppression, resulting in CD8 T-cell malfunction. The Tregs in mice did not express *Ccr5*, so we did not consider them to be direct responders to aPD-1-meidated *Ccl5* upregulation; however, human proliferative Tregs expressed *CCR5*, which implies that Tregs may differ functionally between humans and mice.

We found that aPD-1–mediated *Ccl5* upregulation may be connected to *Ccr5* ^+^ macrophage recruitment. Although *Ccr5* is expressed by multiple cell types, macrophages were strongly affected by aPD-1 treatment. This suggests that *Ccl5* may differentially affect immune cells and might also be regulated by location. *Ccr5* ^+^ macrophages expressed anti-inflammatory genes that were related to the response rate to aPD-1 in a previous study (33). We did not observe distinct upregulation of immunosuppressive cytokines, such as IL-10, in *Ccr5* ^+^ macrophages after aPD-1 treatment, but these macrophages did express elevated levels of *Arg1*. Arg1 is a marker for M2 macrophages that is known to suppress T-cell immunity *via* amino acid deprivation (34, 36). Consistent with that fact, we observed that aPD-1 treatment suppressed transcription, translation, and expression of *Cdk4* and *Ccnd3* in CD8 T cells. These changes are hallmarks of L-Arg starvation. Therefore, we propose increased *Arg1* expression in *Ccr5* ^+^ macrophages as a possible mechanism that induces re-exhaustion of CD8 T cells. This hypothesis must be tested by further research.

We observed re-exhaustion of CD8 T cells after aPD-1 treatment. Although *Pdcd1* expression was reduced by aPD-1, expression of other exhaustion markers, such as *Lag3*, was dramatically increased. This re-expression of exhaustion markers may explain why clinical trials targeting PD-1 have failed. Human data suggested that neoadjuvant PD-1 blockade in patients with rGBM can induce activation of T-cell and cDC1 populations; however, this activation could not overcome the action of immunosuppressive macrophages (17). Their results suggest that additional ligation of other immune checkpoints might inhibit T-cell activation and that TIGIT and CTLA4 may be critical targets for blocking additional exhaustion. Additionally, we propose LAG3 as an attractive target to block along with PD-1. Co-inhibition of LAG3 and PD-1 showed dramatic efficacy in a previous study using a murine GBM model (41).

Our data revealed that aPD-1 increased *Ccl5* expression globally and *Ccr5* expression in macrophages. This implies that aPD-1-induced CCL5 actively recruits CCR5-expressing cells, especially monocytes and macrophages. Previous studies showed that CCL5 expression is associated with better outcomes of aPD-1 therapy due to robust CD8 T-cell recruitment and formation of immunological synapses (42, 43). Our data also show that T cells expressed *Ccr5*, suggesting that CCL5 recruits multiple cells, including T cells and macrophages. According to previous GBM studies, CCL5 recruits CCR5^+^CD38^+^HLA-DR^+^CD8^+^ T cells (44), which showed markers of both exhaustion and activation. CCL5 also recruits macrophages and invasive GBM cells (45). Although the role of the CCL5–CCR5 axis in immunotherapy for GBM is unclear, this axis is related to worse survival of patients and increased resistance to chemotherapy (45, 46). Our data suggest that CCL5 has a dual role, both suppressing and enhancing antitumor immunity. Future studies should clarify these complex roles of CCL5 in the context of GBM because it is possible that selective inhibition of CCR5 in immunosuppressive cells could have clinical benefits.

In summary, we comprehensively analyzed scRNAseq data from an aPD-1-treated GL261-bearing murine GBM model. These data provide important clues to overcome existing hurdles for immunotherapy. We propose that CCL5-mediated immunosuppression may affect re-exhaustion of CD8 T cells through macrophages. In addition, we propose CCL5 and LAG3 as promising target molecules for immunotherapy.

## Materials and methods

### Animals

Eight-week-old specific pathogen-free male C57BL/6J mice were purchased from the DBL. *Cd8* ^−/−^ mice (stock number: 002665, B6S129S2-*Cd8a^tm1Mak^*/J) were purchased from the Jackson Laboratory. All mice used in this study were maintained in a specific pathogen-free facility of the KAIST laboratory Animal Resource Center. Mice were housed in a 12 h/12 h light/dark cycle at 18–24°C with 30%–70% humidity. All procedures for rodent experiments and the determination of end points of animals were performed in accordance with the guidelines and protocols provided by the KAIST Institutional Animal Care and Use Committee (KA2017-41).

### Cell lines

The murine GBM cell line GL261 was kindly gifted by Dr. Injune Kim (KAIST) (47). *Mycoplasma* contamination was tested using an e-Myco plus Mycoplasma PCR Kit (25237; Intro Biotechnology), and no *Mycoplasma* contamination was observed. Cell lines were passaged with trypsin-EDTA (Welgene) and maintained in Dulbecco’s modified Eagle’s medium (RPMI; Corning) with 10% fetal bovine serum (FBS; Welgene) and 1% penicillin-streptomycin (Welgene).

### Tumor models

To induce GBM in the murine model, 1 × 10^5^ GL261 cells in Dulbecco’s phosphate-buffered saline (DPBS) were intracranially injected into the right frontal cortex of isoflurane-anesthetized mice as previously described. Briefly, 1× 10^5^ GL261 cells resuspended in 2μl DPBS were injected 2 mm lateral, 2 mm posterior from the bregma and 3 mm depth at a speed of 0.4μl/min using a stereotaxic apparatus (Stoelting) and an injector (KD Scientific). Upon completing injection, the needle was left in place for 1 min, then withdrawn to help reduce cells reflux. After the injection, the mice were rested separately for the recovery.

To block PD-1, we intraperitoneally injected 200 μg anti-PD-1 antibody (BioXcell) or rat IgG2a isotype control (BioXcell) in 100μl DPBS into mice at 11, 13, and 15 days post tumor-cell injection.

### Cell isolation and flow cytometry

Single-cell suspensions were isolated from tumor tissues as described previously (48). In brief, tumor tissues were harvested and chopped into pieces. Samples were subjected to a mixture of 2 mg/mL Collagenase IV (Worthington) and 30 μg/mL DNase I (Roche) in media for 30 min at 37°C. The samples were then passed through 70-μm strainers (SPL). The resulting cell suspensions were loaded to a 30%–70% Percoll gradient (GE Healthcare) and centrifuged. Red blood cells were depleted by ammonium-chloride-potassium lysis buffer. The cells were then treated with an anti-CD16/32 antibody (2.4G2) to block Fc receptors. Next, the cells were stained using the following antibodies: CD45.2-PE (104; Thermo Fisher Scientific), CD45.2-AF700 (104; Biolegend), CD45.2-BV421 (104; BD Biosciences), CD3e-PE-Cy7 (145-2C11; BD Biosciences), CD4-APC-Cy7 (GK1.5; BD Biosciences), CD4-AF700 (RM4.5; BD Biosciences), CD8a-APC (2.43; TONBO Biosciences), CD8a-APC-Cy7 (53-6.7; Biolegend), CD44-PerCP-Cy5.5 (IM7; Biolegend), CD62L-BV421 (MEL-14; Biolegend), MuLV-tetramer-PE (MBL), CD11b-BV510 (M1/70; Biolegend), and PD-1-PE (J43; BD Biosciences). Live cells were gated based on propidium iodide (PI; Biolegend), 7-aminoactinomycin D (7-AAD; Biolegend), or a Zombie Aqua Fixable Viability Kit (Biolegend). To sort immune cells from tumor tissues, samples were acquired on an FACSAria Fusion (BD Biosciences). Other samples were acquired on an LSRFortessa™ X-20 (BD Biosciences). All data were analyzed with FlowJo (Tree Star).

### Analysis of public data

Public data from human patients were obtained from the CGGA (https://cgga.org.cn) (18). We used RNA-seq data from a total of 325 patients from the CGGA database. We used GEPIA 2 (Gene Expression Profiling Interactive Analysis; https://gepia2.cancer-pku.cn) to analyze TCGA (https://portal.gdc.cancer.gov) data (19). For RNA-seq analysis of patients who received adjuvant or neoadjuvant pemb, we used previously published data (GSE121810) (37). For scRNAseq analysis of patients with rGBM who received neoadjuvant pemb, we used data from a previous study (GSE154795) (17).

### Single-cell transcriptome analysis

At 11, 13, and 15 days post intracranial injection of GL261 cells, mice were injected intraperitoneally with 200μg anti-PD-1 antibody or isotype antibody. At 20 days after tumor-cell injection, single-cell suspensions were isolated and sorted as described previously (48). scRNAseq was performed using a Chromium Single Cell 3’ Reagent kit (10X Genomics) according to the manufacturer’s protocol. A total of 10,000 immune cells were used for analysis. Sequencing results were converted into FASTQ files using Cell Ranger (10X Genomics). Cell sorting and library preparation were carried out by Ms. Jiye Kim at the FACS Core Facility and the NGS Core Facility of the BioMedical Research Center, KAIST. Samples were aligned using the mouse genome (mm10). Data were loaded into Seurat version 4 for analysis. R 4.1.3 was used for statistical analyses. For quality control of samples, cells expressing less than 200 features, more than 6,000 features, and more than 20% mitochondrial genes were excluded. Data were normalized using the NormalizeData function. Variable features were checked by the FindVariableFeature function with the vst method. Then, data from the anti-PD1 group and isotype were integrated by the FindIntegrationAnchors and IntegrateData functions. Then, the integrated data were scaled to center the mean of gene expression level to 0 by the ScaleData function. Dimensions of the integrated and scaled data were reduced using principal component analysis (pca), and 30 significant principal components were used. Data were clustered using the FindNeighbors and FindClusters functions with 0.3 resolution. Data were subjected to the runUMAP function to visualize principal components. We designated clusters using the FindMarkers function and already-known markers. Gene expression was visualized using the DotPlot, VlnPlot, FeaturePlot, and Nebulosa packages (49). Statistical significance of the differentially expressed genes was measured using the stat_compare_means function from the ggpubr package version 0.4.0.

### GO and KEGG enrichment analysis of differentially expressed genes

Using the differentially expressed genes found between anti-PD1 treated group and isotype treated group, enriched pathways and gene sets were analyzed. For pathway analyses, we used ClusterProfiler (50, 51) package version 4.2.2 with the KEGG (22–24) and GO databases (20, 21) by using the enrichKEGG and enrichGO function, respectively. A corrected P value<0.05 (padj < 0.05) was considered statistical significantly.

### Gene-set enrichment analysis of differentially expressed genes

Gene set enrichment analysis v4.2.3 (GSEA; Broad Institute) was done using the deferentially expressed genes to find enriched gene sets related to the biological pathways based on the MSigDB 7.0 (52, 53). Only the pathway with FDR <0.05 was considered statistical significantly.

### Statistical analysis

Data are expressed as mean ± standard error of the mean. Differences among groups were analyzed by unpaired, two-tailed Student’s *t* test or Mann–Whitney U test. The log-rank test was used for survival data. To analyze correlation data, we used Spearman’s correlation test. Statistical analysis was performed using R software (R Foundation for Statistical Computing) or Prism software (GraphPad). Differences were considered statistically significant at *P* < 0.05.

## Data availability statement

The datasets presented in this study can be found in online repositories. The names of the repository/repositories and accession number(s) can be found below: https://www.ncbi.nlm.nih.gov/geo/, GSE154795.

## Ethics statement

The animal study was reviewed and approved by KAIST Institutional Animal Care and Use Committee.

## Author contributions

JP, IK, and HL designed and conducted the experiments, analyzed the data, and wrote the manuscript. All authors contributed to the article and approved the submitted version.
