# Supplementary material for: The immune landscape of high-grade brain tumor after treatment with immune checkpoint blockade
Source: Front Immunol. 2022 Dec 14;13:1044544. doi: 10.3389/fimmu.2022.1044544 (PMC9794569; doi:10.3389/fimmu.2022.1044544)
Supplement: Supplementary file 1 [file DataSheet_1.pdf]

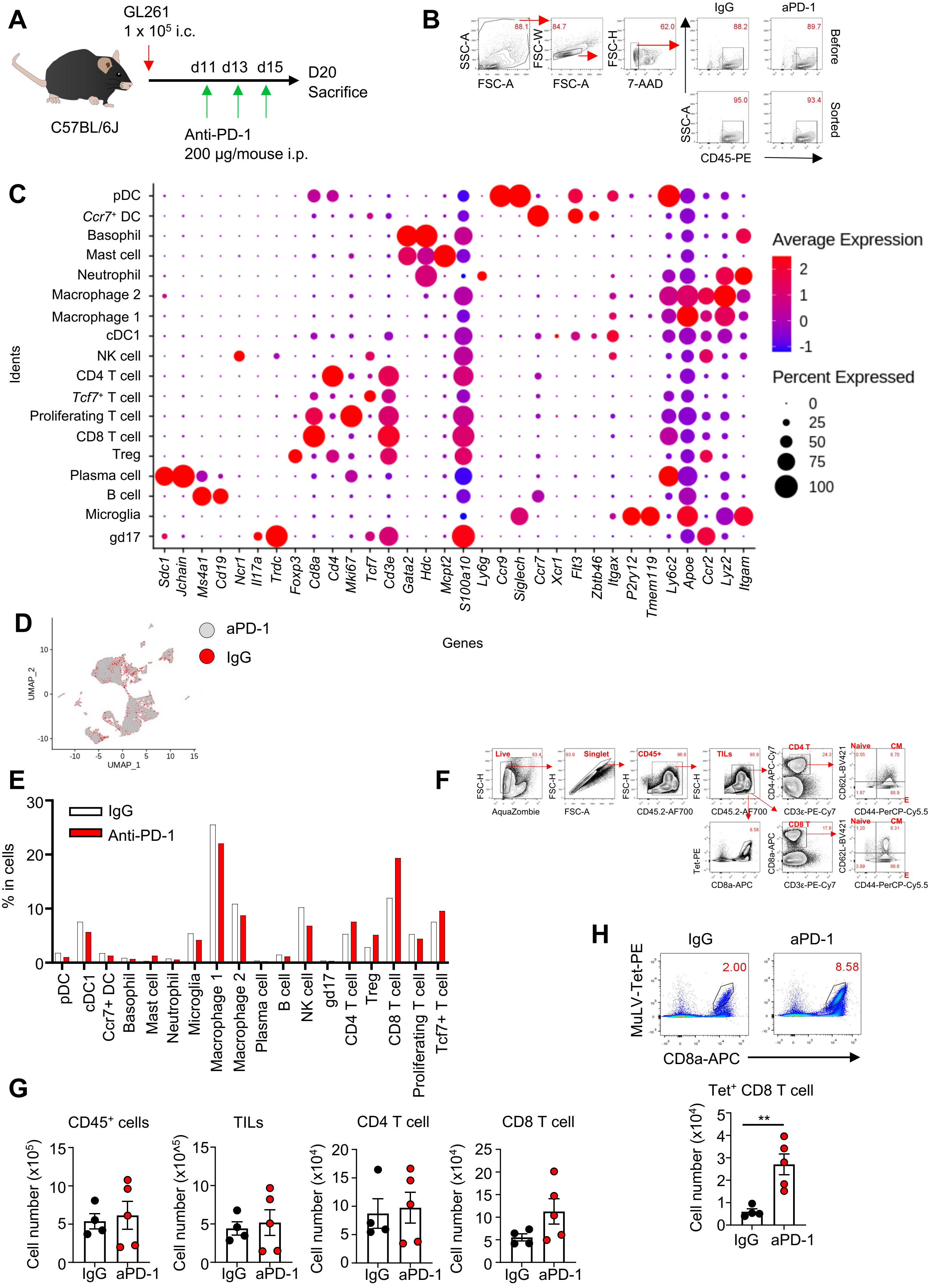

Park and Kang, Figure S1

Figure S1. Immune reaction to aPD-1 treatment in GL261-injected mice, related to Figure 1.

(**A**) Experimental scheme. Eight-week-old male C57BL/6J mice were intracranially injected with GL261 cells. At 11, 13, and 15 days post tumor injection, 200 μg aPD-1 or IgG control was intraperitoneally injected into the mice. At 20 days post tumor injection, the mice were sacrificed and analyzed. (**B**) Gating strategy for immune cell sorting from tumor tissues. (**C**) Expression of marker genes for identification of clusters. (**D**) UMAPs of cells from IgG- and aPD-1-treated mice were overlapped. (**E**) Frequencies of clusters from IgG- or aPD-1-treated groups were compared. (**F–H**) At 20 days post tumor injection, IgG- or aPD-1-treated mice were sacrificed, and cells were isolated and analyzed by flow cytometry. (**F**) Gating strategy. (**G**) The numbers of $CD45^{+}$ cells, tumor-infiltrating leukocytes (TILs; $CD45^{hi}$), CD4 T cells ($CD45^{hi}CD3e^{+}CD4^{+}$), and CD8 T cells ($CD45^{hi}CD3e^{+}CD8a^{+}$) were analyzed. (**H**) MuLV tetramer-specific CD8 T cells were analyzed. Gating (upper) and the number of tetramer-specific CD8 T cells (below) are shown. Data in **G** and **H** were analyzed by unpaired, two-tailed Student's *t* test. *$P<0.05$, **$P<0.01$. Error bars represent the mean ± standard error of the mean (SEM).

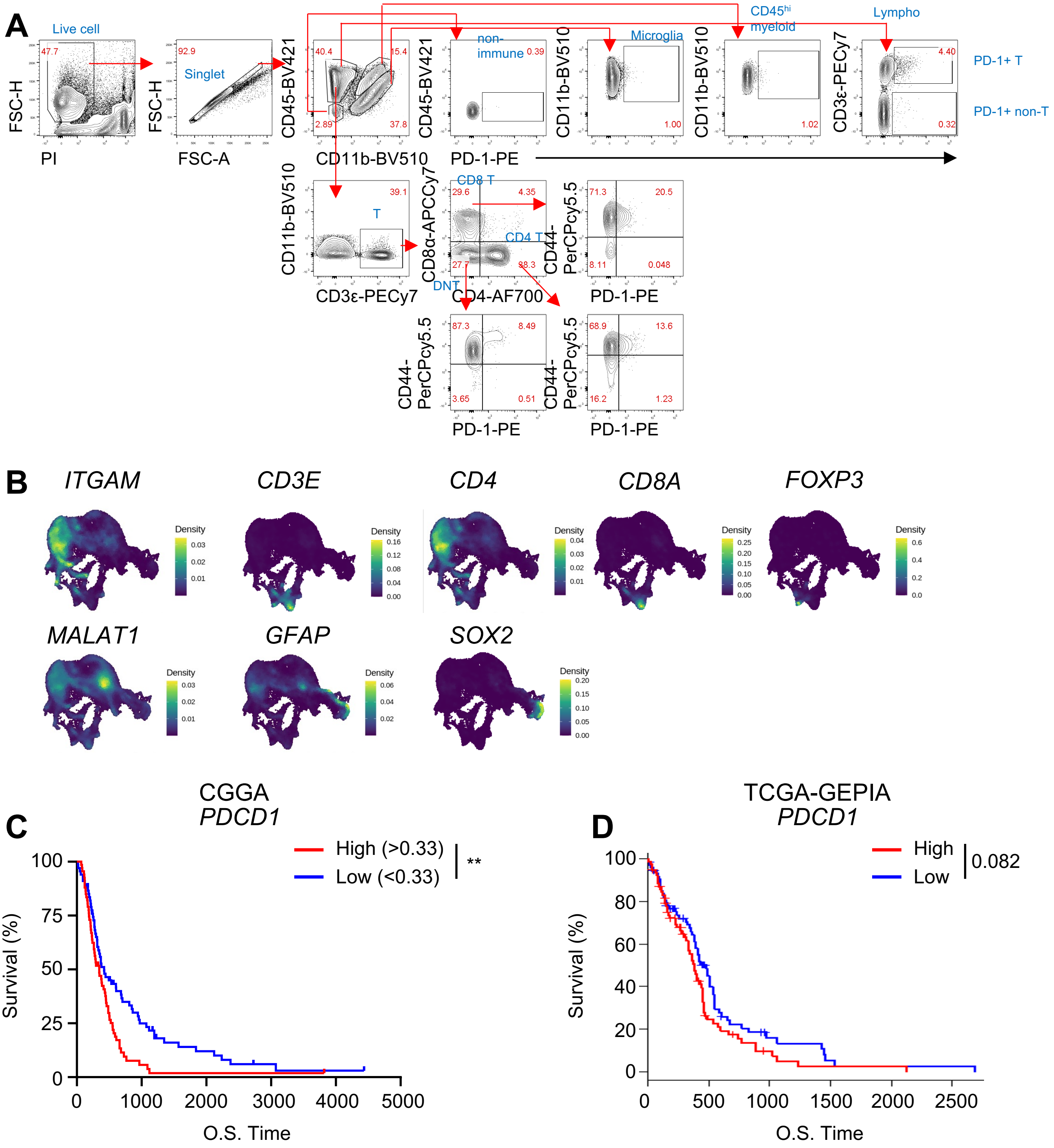

Park and Kang, Figure S2

Figure S2. PD-1 expression in immune cells from GBM tissues, related to Figure 2.

(**A**) Gating strategy for Figure 2. (**B**) Expression density of *ITGAM*, *CD3E*, *CD4*, *CD8A*, *FOXP3*, *MALAT1*, *GFAP*, and *SOX2* from scRNAseq data GSE154795. (**C**) Overall survival of patients with GBM from the CGGA database divided by *PDCD1* expression (divided by median value of 0.33; 69 patients for the low group and 67 patients for the high group). (**D**) Overall survival of patients with GBM from the GEPIA database divided by *PDCD1* expression (divided by median value; 80 patients for the high group and 78 patients for the low group). Survival data were analyzed by log-rank test. *P<0.05, **P<0.01

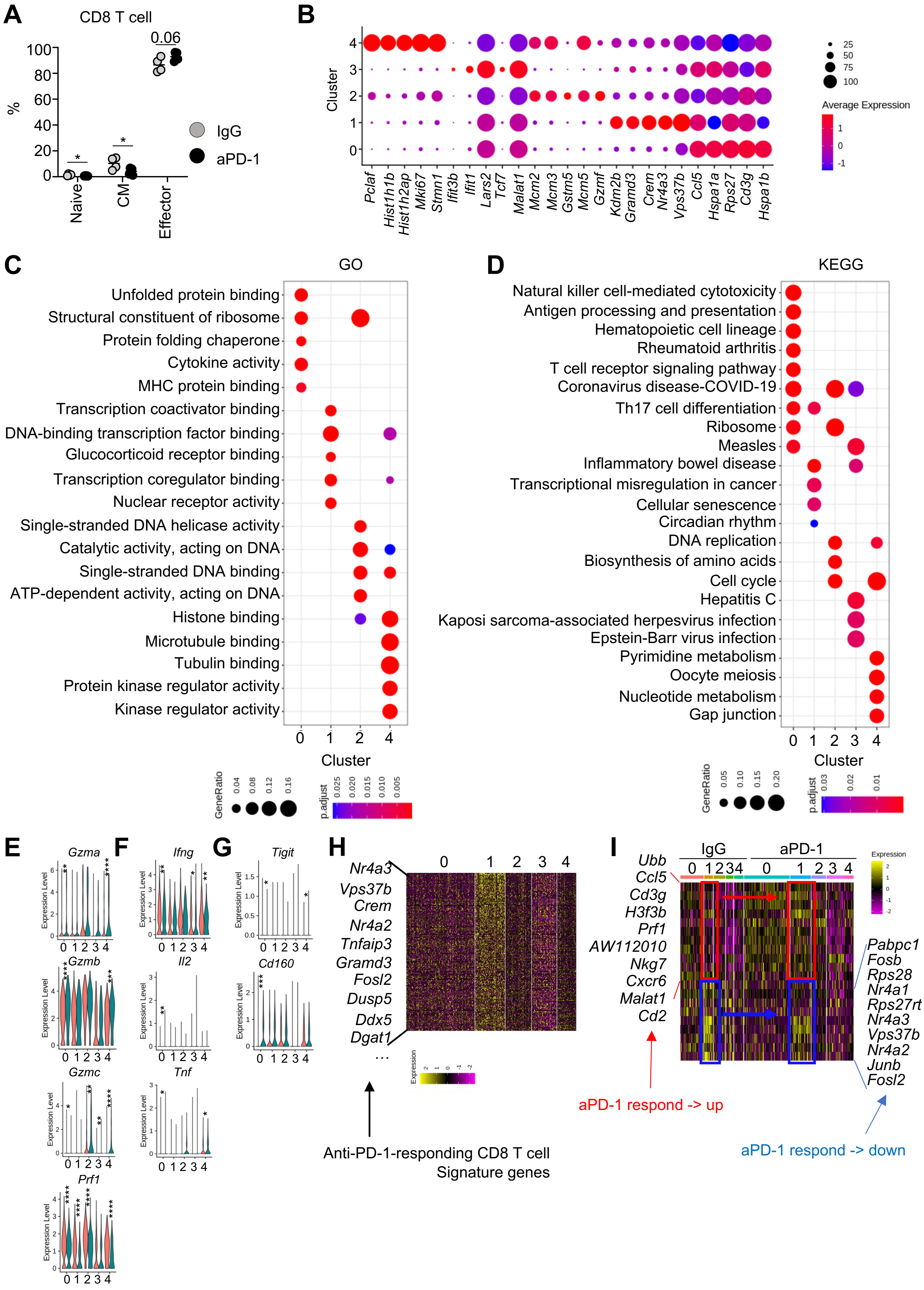

Park and Kang, Figure S3

Figure S3. The landscape of CD8 T cells in brain tumors, related to Figure 3.

(**A**) Eight-week-old male C57BL/6J mice were intracranially injected with GL261 cells. After three treatments with aPD-1, at 20 days post tumor injection, cells were isolated and analyzed by flow cytometry. The percentages of naïve ($CD44^{-}CD62L^{+}$), central memory (CM; $CD44^{+}CD62L^{+}$), and effector ($CD44^{+}CD62L^{-}$) cells among the CD8 T cells were analyzed. (**B–I**) CD8 T cells ($Cd3e^{+}Cd8a^{+}Cd4^{-}$doublet^{-}) were isolated from scRNAseq data and analyzed. (**B**) Dot plot for marker genes of CD8 T-cell clusters. (**C, D**) DEGs of clusters were analyzed to find enriched pathways using the GO (**C**) and KEGG (**D**) databases. (**E-G**) The expression of *Gzma*, *Gzmb*, *Gzmc*, *Prf1* (**E**), *Ifng*, *Il2*, *Tnf* (**F**), *Tigit*, and *Cd160* (**G**). (**H**) DEGs from cluster 1 compared with others were designated as "anti-PD-1-responding CD8 T-cell signature genes" and visualized by heatmap. (**I**) DEGs of cluster 1 from the aPD-1 group (red) and the IgG group (blue) were visualized by heatmap. Data in **A** were analyzed by unpaired, two-tailed Student's *t* test. Data in **E–G** were analyzed by the stat_compare_means function with unpaired t-test. *$P<0.05$. Error bars represent the mean ± standard error of the mean (SEM).

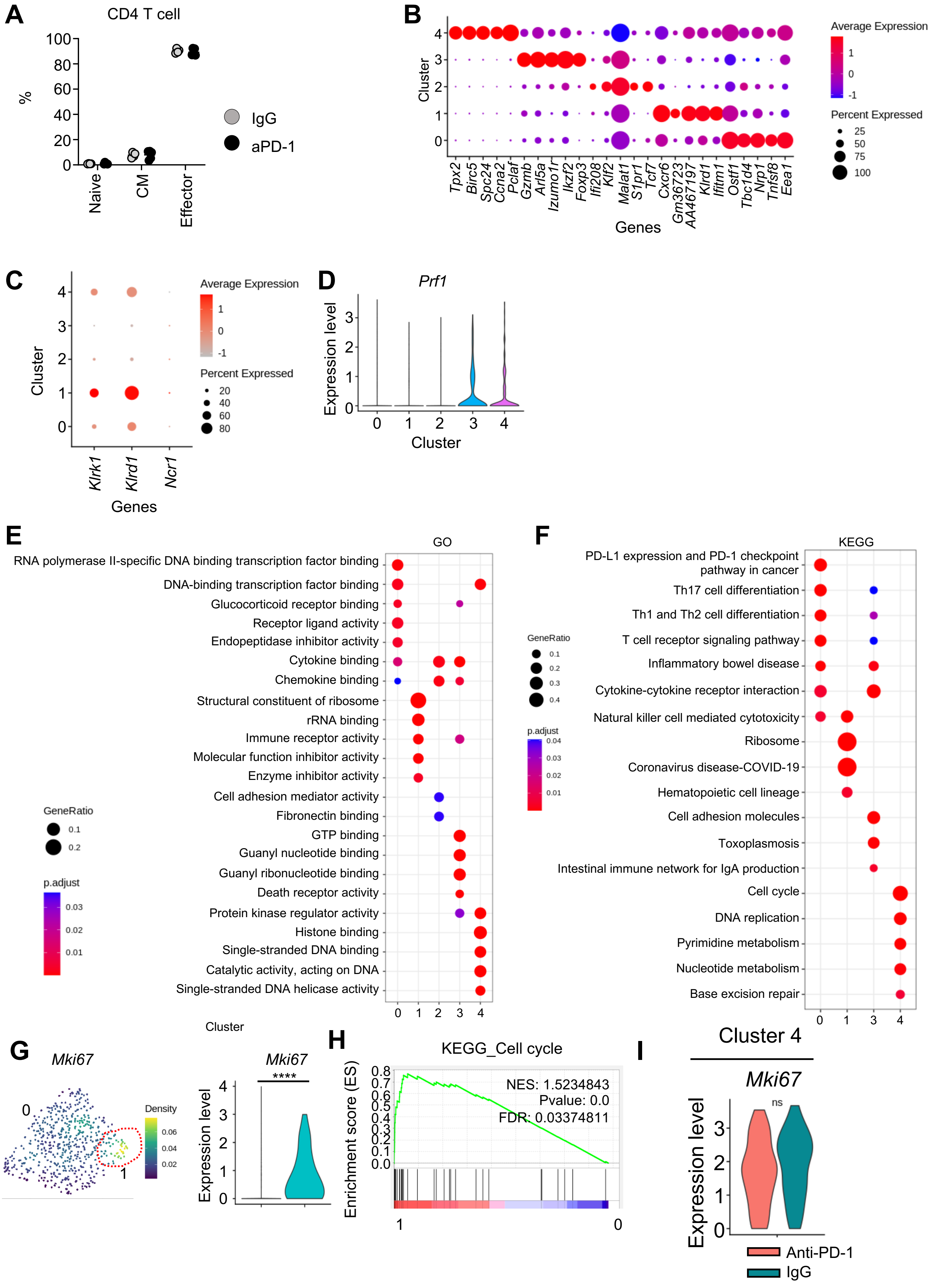

Park and Kang, Figure S4

Figure S4. The CD4 T-cell transcriptome in brain tumors, related to Figure 4.

(**A**) Eight-week-old male C57BL/6J mice were intracranially injected with GL261 cells. After three treatments with aPD-1, cells were isolated and analyzed by flow cytometry at 20 days post tumor injection. The percentages of naïve ($CD44^{-}CD62L^{+}$), central memory (CM; $CD44^{+}CD62L^{+}$), and effector ($CD44^{+}CD62L^{-}$) cells among the CD4 T cells were analyzed. (**B–F**) CD4 T cells ($Cd3e^{+}Cd4^{+}Cd8a^{-}$doublet^{-}) were isolated from scRNAseq data and analyzed. (**B**) Marker genes of clusters are displayed by dot plot. (**C**) Expression of *Klrk1*, *Klrd1*, and *Ncr1* from CD4 T-cell clusters was analyzed. (**D**) *Prf1* expression from clusters was analyzed. (**E, F**) Enriched pathways from CD4 T-cell clusters were analyzed based on the GO (**E**) and KEGG (**F**) databases. (**G**, **H**) Tregs ($Cd3e^{+}Cd4^{+}Foxp3^{+}$) were isolated from scRNAseq data. (**G**) *Mki67* expression density and level were analyzed. (**H**) DEGs from Treg cluster 1 compared to Treg cluster 0 were analyzed using KEGG_Cell cycle gene sets. (**I**) The *Mki67* expression level of CD4 T cell cluster 4 was analyzed. Data in **A** were analyzed by unpaired, two-tailed Student's *t* test. Data in **G** and **I** were analyzed by the stat_compare_means function with unpaired t-test. *$P<0.05$, **$P<0.01$, ***$P<0.001$, ****$P<0.0001$. Error bars represent the mean ± standard error of the mean (SEM). NES: normalized enrichment score; FDR: false discovery rate.

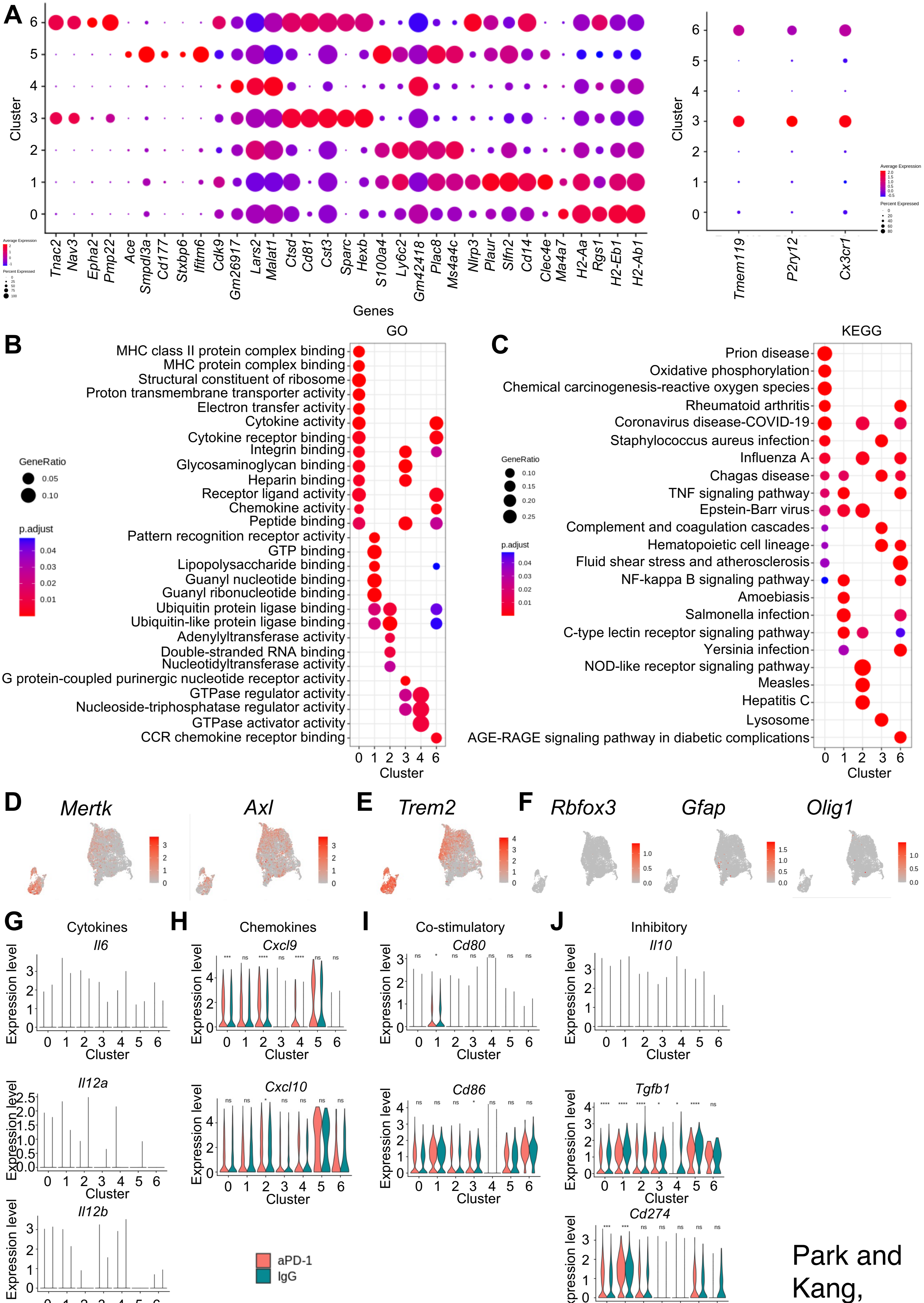

Park and Kang, Figure S5

Figure S5. Transcriptomes of microglia and macrophages in the brain tumor tissue, related to Figure 5.

(**A**) Microglia and macrophage clusters were isolated from scRNAseq data. After re-clustering, marker genes of clusters were analyzed. (**B-C**) Enriched pathways based on DEGs of clusters were analyzed using the GO (**B**) and KEGG (**C**) databases. (**D–F**) Expression of *Mertk* and *Axl* (**D**), *Trem2* (**E**), and *Rbfox3*, *Gfap*, and *Olig1* (**F**) is shown by feature plots. (**G–J**) Expression of cytokines (*Il6*, *Il12a*, *Il12b*) (**G**), chemokines (*Cxcl9*, *Cxcl10*) (**H**), co-stimulatory genes (*Cd80*, *Cd86*) (**I**), and inhibitory genes (*Il10*, *Tgfb1*, *Cd274*) (**J**) from macrophage/microglia clusters was analyzed. Data were analyzed by the stat_compare_means function with unpaired t-test. *P<0.05, **P<0.01, ***P<0.001, ****P<0.0001.

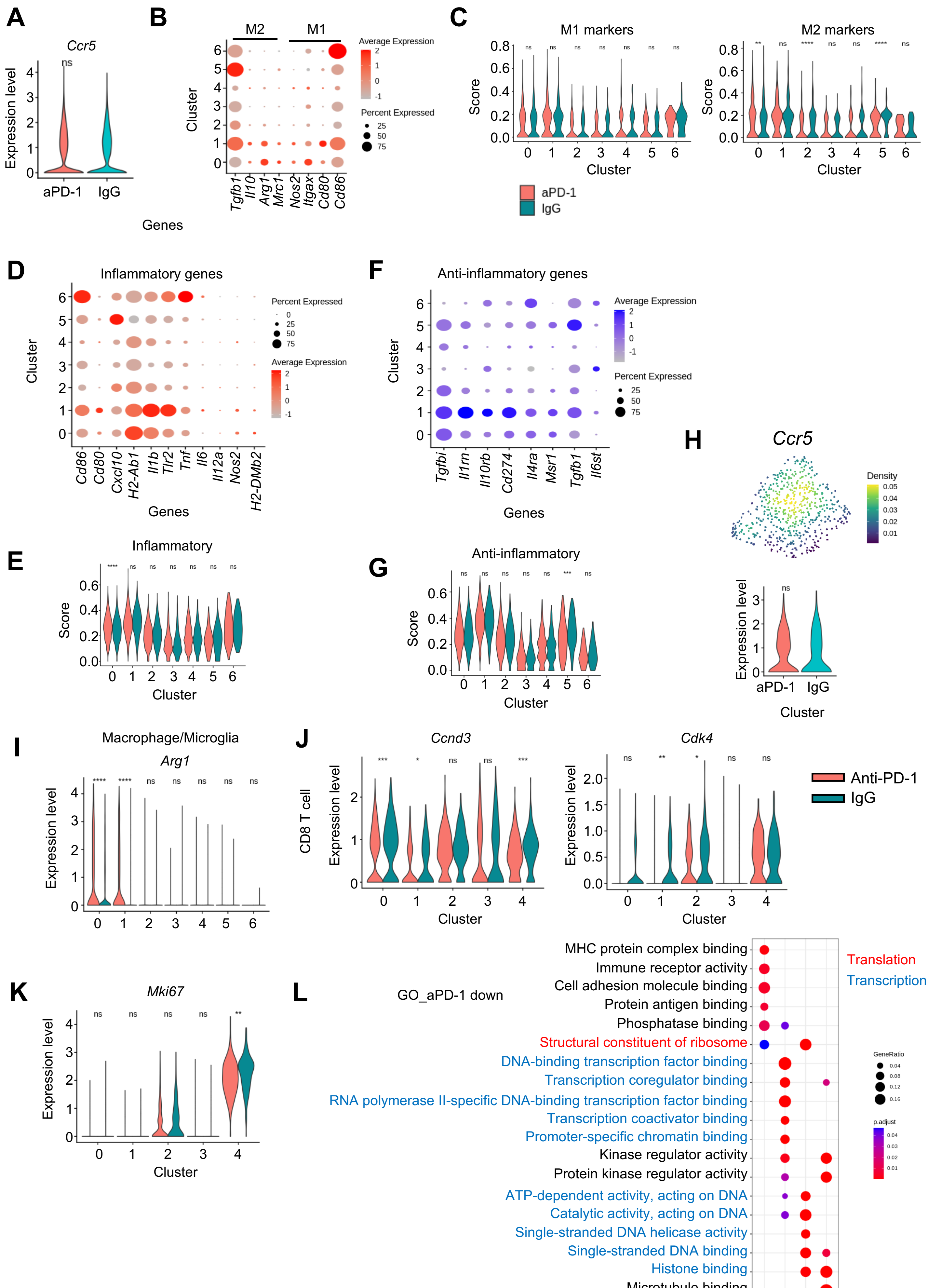

Park and Kang, Figure S6

Figure S6. Immunosuppressive effects of aPD-1 treatment, related to Figure 6.

(**A**) Expression of *Ccr5* in total cells from scRNAseq data was analyzed. (**B, C**) Expression level (**B**) and score (**C**) of M1 and M2 markers from macrophage/microglia subclusters. (**D–E**) Expression level (**D, F**) and score (**E, G**) of inflammatory (**D, E**) and anti-inflammatory (**F, G**) genes in macrophage/microglia subclusters. (**H**) *Ccr5* expression density and level in Treg subclusters were analyzed. (**I**) Expression of *Arg1* in macrophage/microglia subclusters. (**J–K**) Expression of *Ccnd3* and *Cdk4* (**J**) and *Mki67* (**K**) in CD8 T-cell subclusters was analyzed. (**L**) Pathways enriched in CD8 T-cell subclusters from IgG-treated mice compared with aPD-1-treated mice were analyzed using the GO database. Translation-related pathway (red), transcription-related pathways (blue), and other pathways (black). Data were analyzed by the stat_compare_means function with t-test. *P<0.05, **P<0.01, ***P<0.001, ****P<0.0001.

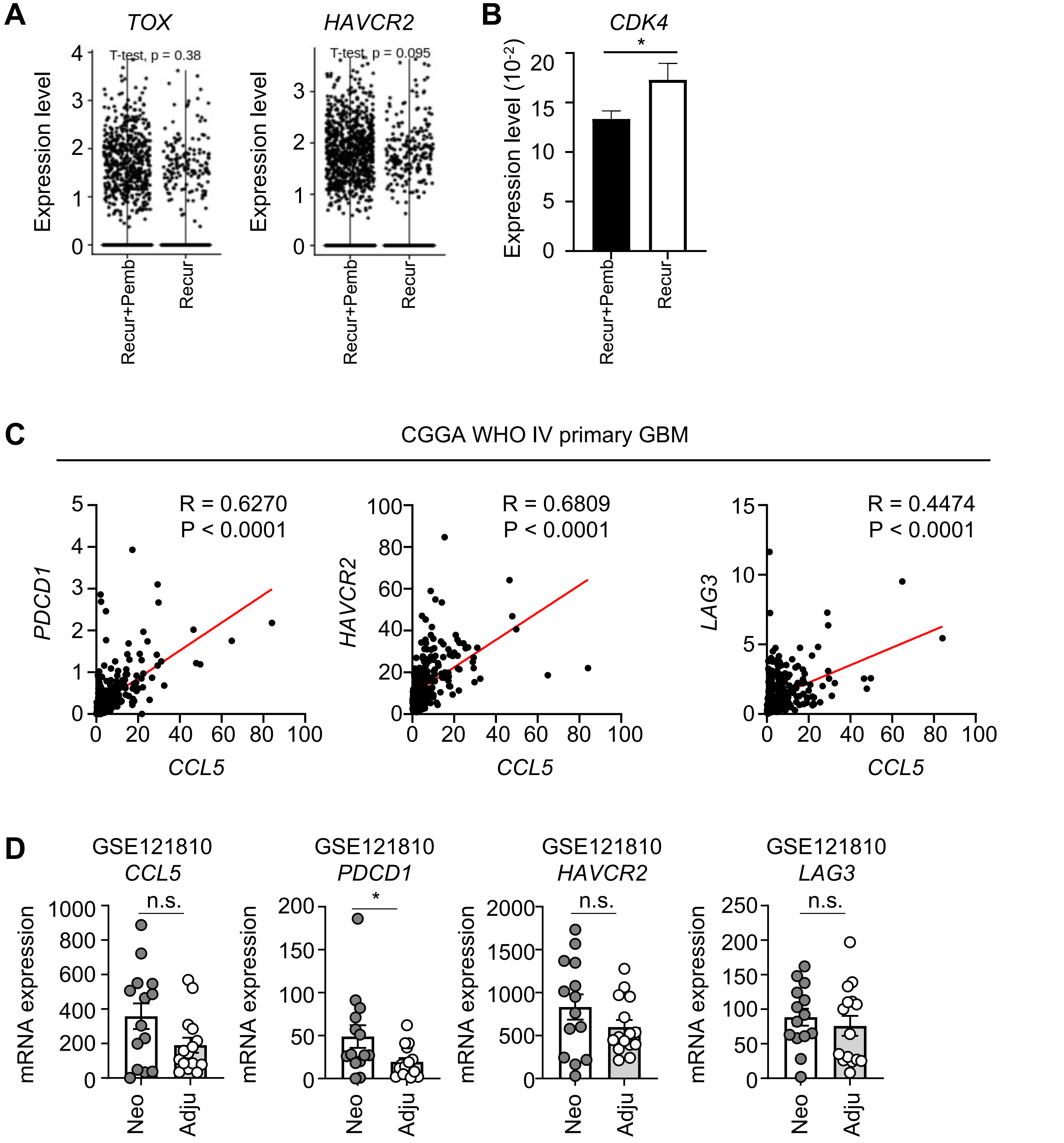

Park and Kang, Figure S7

Figure S7. The effect of aPD-1 in human patients, related to Figure 7.

(**A**) Expression of *TOX* and *HAVCR2* in CD8 T-cell clusters of the GSE154795 data set. (**B**) Expression of *CDK4* in CD8 T-cell clusters of the GSE154795 dataset. (**C**) Using WHO grade IV primary GBM patient data from the CGGA database, correlations between *CCL5* and *PDCD1*, *HAVCR2*, and *LAG3* were analyzed. (**D**) Using RNAseq GSE121810 data, expression of *CCL5*, *PDCD1*, *HAVCR2*, and *LAG3* was compared between neoadjuvant and adjuvant pembrolizumab-treated samples. Data in **A** were analyzed by the stat_compare_means function with t-test. Data in **B** and **D** were analyzed by unpaired, two-tailed Student's *t* test. Data in **C** were analyzed by Spearman correlation test. *P<0.05, **P<0.01, ***P<0.001, ****P<0.0001. Error bars represent the mean ± standard error of the mean (SEM).

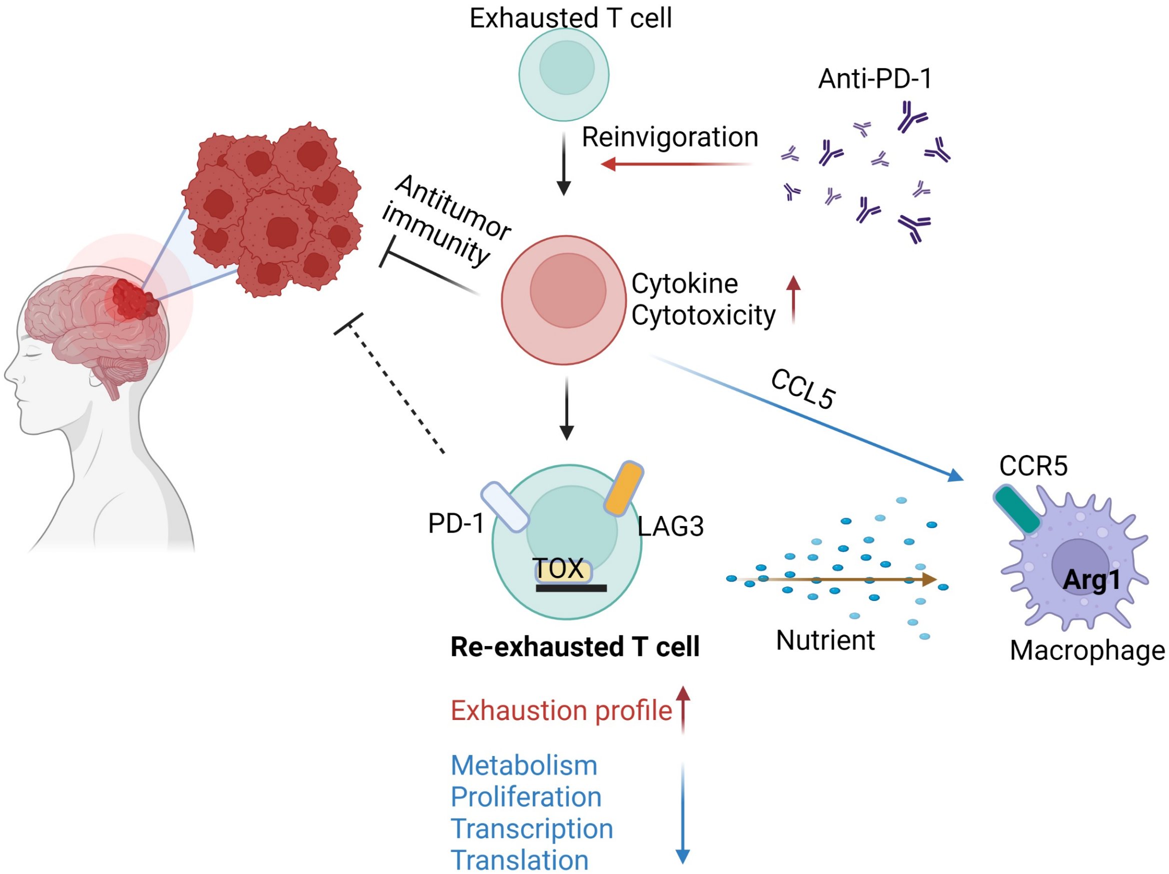

Park and Kang, Figure S8

Figure S8. Scheme of mechanisms.

Within the brain tumor microenvironment, T cells are exhausted. If anti-PD-1 therapy is given, CD8 T cells can be reinvigorated by upregulation of cytokines and cytotoxicity molecules. Reinvigorated T cells also express CCL5, which recruits macrophages. $CCR5^{+}$ macrophages are anti-inflammatory and hijack nutrients from T cells via Arg1. As a result, T cells are re-exhausted. Re-exhausted T cells are marked by expression of PD-1, LAG3, and TOX. These cells show increased exhaustion profiles as pathways related to metabolism, proliferation, transcription, and translation are downregulated. This image was created with BioRender.com
